# Copper‐Doped Prussian Blue Nanozymes With Hyaluronic Acid‐Mediated Targeting Alleviate Oxidative Stress and Regulate Cholesterol Handling for Atherosclerosis Therapy

**DOI:** 10.1002/advs.76976

**Published:** 2026-08-05

**Authors:** Jianliang Ou, Ruihua Ji, Qinglu Zang, Mingkang Wang, Lingling Zhou, Yingliang Wei, Di Yang, Yue Wu, Hongxu Zhu, Jianrong Wu, Xiaojun Cai, Wenxian Du, Junjie Cheng, An Zeng, Yuehua Li

**Affiliations:** ^1^ Department of Radiology Shanghai Jiao Tong University School of Medicine Affiliated Sixth People's Hospital Shanghai People's Republic of China; ^2^ Department of Anesthesiology Changzheng Hospital Naval Medical University Shanghai People's Republic of China; ^3^ College of Chemistry and Materials Science Shanghai Normal University Shanghai People's Republic of China; ^4^ Wuhan United Imaging Life Science Instruments Ltd. Wuhan People's Republic of China; ^5^ Faculty of Medical Imaging Technology College of Health Science and Technology Shanghai Jiao Tong University School of Medicine Shanghai People's Republic of China; ^6^ Shanghai Normal University College of Life Science Shanghai People's Republic of China; ^7^ Key Laboratory of Multi‐Cell Systems Institute of Biochemistry and Cell Biology Shanghai Institutes for Biological Sciences Chinese Academy of Sciences Center for Excellence in Molecular Cell Science University of the Chinese Academy of Sciences Chinese Academy of Sciences Shanghai People's Republic of China; ^8^ Department of Pharmacy School of Medicine Renji Hospital Shanghai Jiao Tong University Shanghai People's Republic of China; ^9^ Department of Ultrasound in Medicine Shanghai Jiao Tong University School of Medicine Affiliated Sixth People's Hospital Shanghai People's Republic of China; ^10^ Department of Nutrition and Food Hygiene School of Public Health Southeast University Nanjing People's Republic of China

**Keywords:** ABCA1, ABCG1, CD44, cell biology, chemistry, crosstalk, inflammation, internalization, macrophage, oxidative stress

## Abstract

Atherosclerosis is driven by the persistent crosstalk among chronic inflammation, oxidative stress, and lipid dysmetabolism, largely orchestrated by plaque‐resident macrophages. However, therapeutic strategies capable of simultaneously modulating these interconnected pathological processes are still limited. Herein, we developed a hyaluronic acid‐coated, copper‐doped Prussian blue nanozyme (CuPB@HA) as a CD44‐associated plaque‐targeted nanotherapeutic. Guided by transcriptomic evidence of CD44 enrichment in atherosclerotic plaque macrophages, HA was incorporated to enhance lesion targeting and cellular internalization. In ox‐LDL‐stimulated macrophages, CuPB@HA effectively alleviated oxidative stress, suppressed inflammation, and attenuated lipid accumulation. Mechanistically, it reduced CD36‐dependent lipid uptake and increased the expression of cholesterol‐efflux‐related transporters ABCA1 and ABCG1, thereby shifting macrophages away from a pro‐inflammatory phenotype. In vivo, CuPB@HA preferentially accumulated within atherosclerotic lesions of ApoE^−/−^ mice, significantly reducing plaque burden and improving plaque stability‐associated histological features. Collectively, CuPB@HA integrates redox regulation, macrophage lipid‐handling modulation, and inflammation attenuation, highlighting its potential as a targeted therapeutic strategy for atherosclerosis.

## Graphical abstract

CuPB@HA is a CD44‐associated, plaque‐targeted nanozyme that alleviates oxidative stress, inflammation, and lipid accumulation in macrophages. By reducing CD36‐dependent lipid uptake and promoting ABCA1/ABCG1‐mediated cholesterol handling, it improves macrophage function, preferentially accumulates in atherosclerotic lesions, reduces plaque burden, and enhances plaque stability in ApoE^−/−^ mice through targeted redox regulation mechanisms.

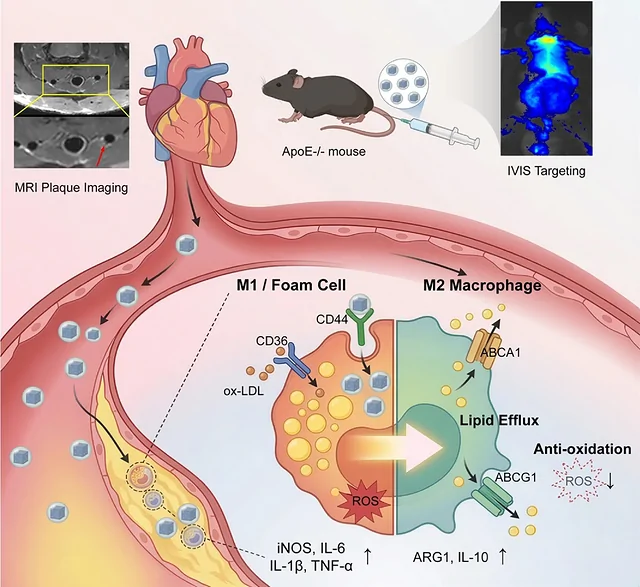

## 1 Introduction

Atherosclerosis (AS) is the leading cause of cardiovascular morbidity and mortality worldwide. It is fundamentally a progressive vascular disease driven by the complex interplay between lipid metabolism disorders and chronic inflammation [1, 2]. During the evolution of atherosclerotic plaques, macrophage dysfunction serves as the cornerstone of disease pathogenesis [3]. Following monocyte infiltration into the intima and subsequent differentiation into macrophages, these cells undergo unrelenting lipid uptake, ultimately transforming into lipid‐engorged foam cells [4]. This transformation triggers macrophage polarization toward a pro‐inflammatory M1 phenotype, leading to the massive secretion of pro‐inflammatory cytokines—such as IL‐6, IL‐1β, and IL‐12—which further exacerbates local oxidative stress [5, 6]. Conversely, anti‐inflammatory M2 macrophages contribute to plaque stabilization [7]. Although systemic lipid‐lowering therapies, such as statins, have achieved significant clinical efficacy, considerable residual cardiovascular risks and potential adverse drug reactions remain [8, 9]. Therefore, there is an urgent need to develop targeted therapeutic strategies capable of simultaneously modulating macrophage polarization, alleviating oxidative stress, and improving macrophage cholesterol‐handling programs for the effective intervention of AS.

Overcoming the limitations of traditional systemic therapies hinges on the precise identification of therapeutic targets within the lesional microenvironment. During the initiation and progression of atherosclerosis, accelerated extracellular matrix (ECM) remodeling is intricately linked to local chronic inflammation [10]. Hyaluronic acid (HA), a critical ECM component, is significantly enriched in human primary and secondary atherosclerotic plaques, where it also exerts immunomodulatory functions [11]. HA possesses a naturally high binding affinity for receptors such as RHAMM (receptor for HA‐mediated motility), Lyve‐1, and CD44 [12]. Consequently, nanodelivery systems engineered with HA can offer the dual advantages of high biocompatibility and low immunogenicity, while achieving specific accumulation in CD44‐overexpressing cells. This targeted approach lays a crucial foundation for the precise remodeling of the plaque microenvironment.

Once precise accumulation at the lesion site is achieved, effectively reversing the severe oxidative stress and metabolic imbalance within the plaque remains a formidable challenge. Nanomaterials exhibiting intrinsic enzyme‐like catalytic activities (i.e., nanozymes) have emerged as promising candidates for scavenging reactive oxygen species (ROS) [13, 14]. Prussian blue (PB) nanoparticles, a classic biocompatible material, possess both superoxide dismutase (SOD)‐ and catalase (CAT)‐mimicking activities [15, 16]. However, metal‐based nanozymes often suffer from suboptimal catalytic efficiency in complex in vivo environments. Doping the PB lattice with transition metals, such as copper (Cu), not only significantly enhances its cascade catalytic capacity for the highly efficient elimination of excess ROS but also endows the material with novel biological functionalities [17, 18]. Furthermore, copper ions serve as indispensable cofactors for various metabolic enzymes and play a distinct role in regulating cellular lipid transport and macrophage polarization [19, 20]. Thus, the synergistic integration of potent ROS‐scavenging capabilities with the metabolic regulatory potential of copper ions holds immense promise for profoundly halting, or even reversing, the pathological progression of atherosclerosis.

In this study, we aimed to engineer a hyaluronic acid‐coated, copper‐doped Prussian blue nanozyme (CuPB@HA) for the targeted therapy of atherosclerotic lesions (Figure 1). This nanozyme orchestrates the remodeling of the lesional macrophage microenvironment through the synergistic effects of HA‐mediated precision targeting and the robust catalytic activity of CuPB. Both in vitro and in vivo studies demonstrate that, upon targeting lesional macrophages, CuPB@HA exhibits exceptional ROS‐scavenging efficacy within the local microenvironment, successfully curbs abnormal lipid influx, and reactivates the reverse cholesterol transport network. Concurrently, the inflammatory cascades within the lesion are significantly reprogrammed, driving the microenvironment toward a pro‐resolving state. This study not only highlights the unique advantages of metal‐doped nanozymes in regulating the “lipid‐inflammation” axis but also provides a highly promising and translatable nanomedicine paradigm for the precision intervention of atherosclerosis.

**FIGURE 1:**
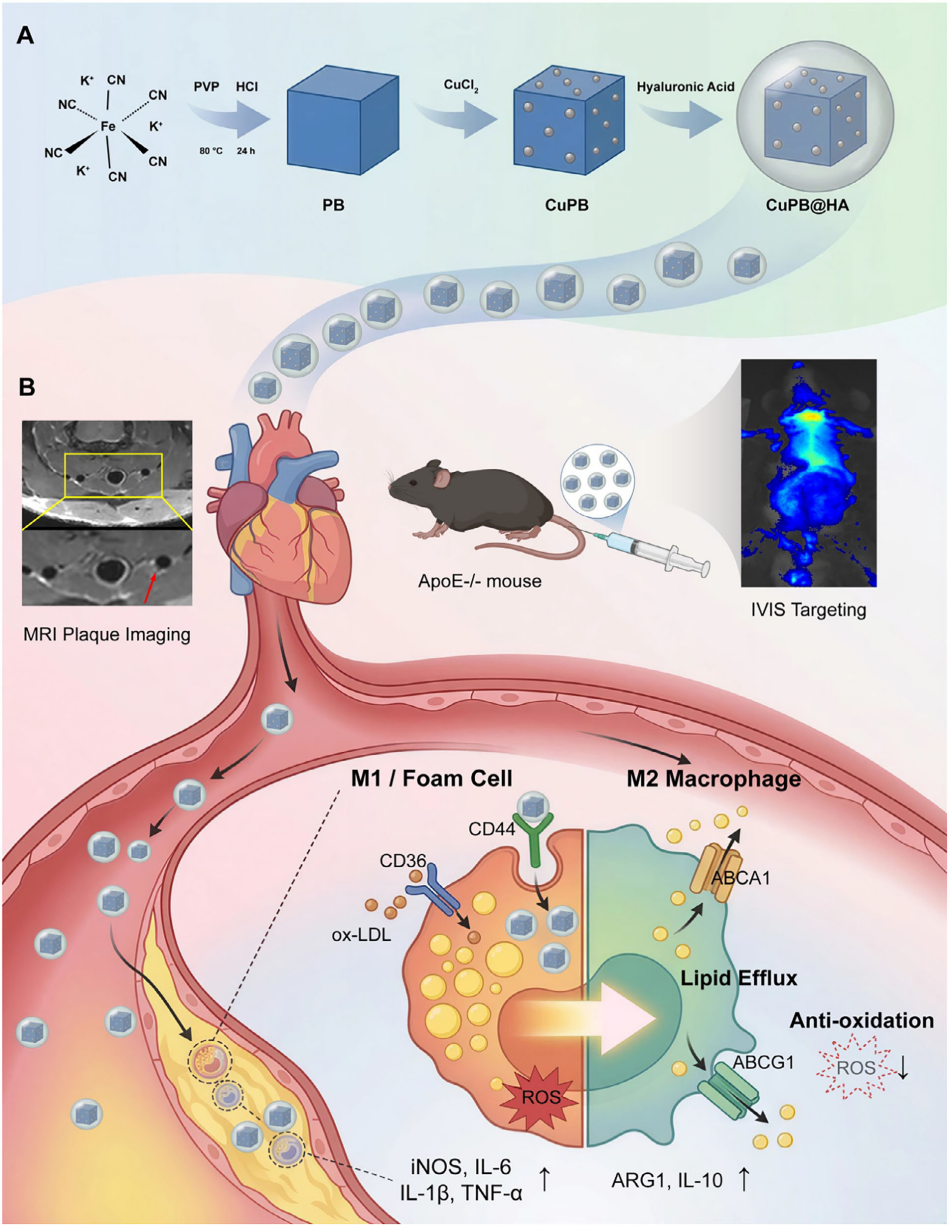
Preparation and anti‐atherosclerotic mechanisms of the CuPB@HA nanozyme. (A) Synthesis procedure of CuPB@HA. PB nanoparticles were prepared through a PVP/HCl‐assisted thermal reaction using K_3_[Fe(CN)_6_] as the precursor, followed by Cu incorporation to obtain CuPB and subsequent HA functionalization to form CuPB@HA. (B) Therapeutic mechanisms. Systemically administered CuPB@HA selectively targets lesional CD44^+^ macrophages. Upon internalization, it synergistically remodels the plaque microenvironment by scavenging ROS to promote a shift toward an anti‐inflammatory macrophage phenotype and improving macrophage lipid‐handling profiles by downregulating CD36 and upregulating ABCA1/ABCG1‐related cholesterol transport mediators, thereby attenuating foam‐cell lipid accumulation. Some elements in the image were sourced from BioRender (https://app.biorender.com/illustrations/69c95a7d8bdf29a2ebf6bea4).

## 2 Result

### 2.1 Single‐Cell Transcriptomics Reveals CD44 Enrichment on Pathogenic Macrophages in Symptomatic Atherosclerotic Plaques

We analyzed a public single‐cell RNA sequencing (scRNA‐seq) dataset of human carotid atherosclerotic plaques (GSE253903) to delineate the intralesional cellular landscape and identify targetable pathogenic subsets. Following quality control, a total of 52,436 single cells from 12 subjects (6 symptomatic and 6 asymptomatic) were retained. Uniform Manifold Approximation and Projection (UMAP) and canonical marker profiling delineated the major immune and stromal cell lineages within the plaque (Figure 2A,B). Feature expression analysis revealed that CD44 transcripts were primarily enriched in macrophages, T and NK cells, fibroblasts, smooth muscle cells, and mast cells (Figure 2C). Exploratory profiling across all 12 major cell populations further illustrated the distribution pattern of CD44 expression between symptomatic and asymptomatic plaques across multiple lineages (Figure S1). To further validate the relevance of CD44 in atherosclerotic lesions and macrophage‐derived foam cells, we performed in vivo and in vitro validation experiments. Immunofluorescence staining showed increased CD44 expression in aortic root plaques from HFD‐fed ApoE^−/−^ mice compared with corresponding arterial tissues from C57BL/6 control mice. Consistently, Western blot analysis showed that ox‐LDL‐induced RAW264.7 foam cells exhibited higher CD44 protein expression than untreated control cells, with densitometric quantification showing an increase from 0.872 ± 0.044 to 1.419 ± 0.158, corresponding to an approximately 1.63‐fold increase (Figure S2). We then evaluated the expression density of functional genes to localize intralesional pathogenesis. Transcript density plots highlighted that the key mediators driving inflammation, oxidative stress, and lipid metabolism were specifically distributed within the macrophage population (Figure S3). More importantly, joint kernel density mapping directly visualized and verified the overlapping expression patterns between CD44 and these functional markers (Figure S4). Consequently, combined with the finding that cell‐level visualization showing CD44 enrichment in plaque macrophages (Figure 2D), we determined that CD44 expression levels on macrophages are plaque‐associated target linked to macrophage‐rich inflammatory regions.

**FIGURE 2:**
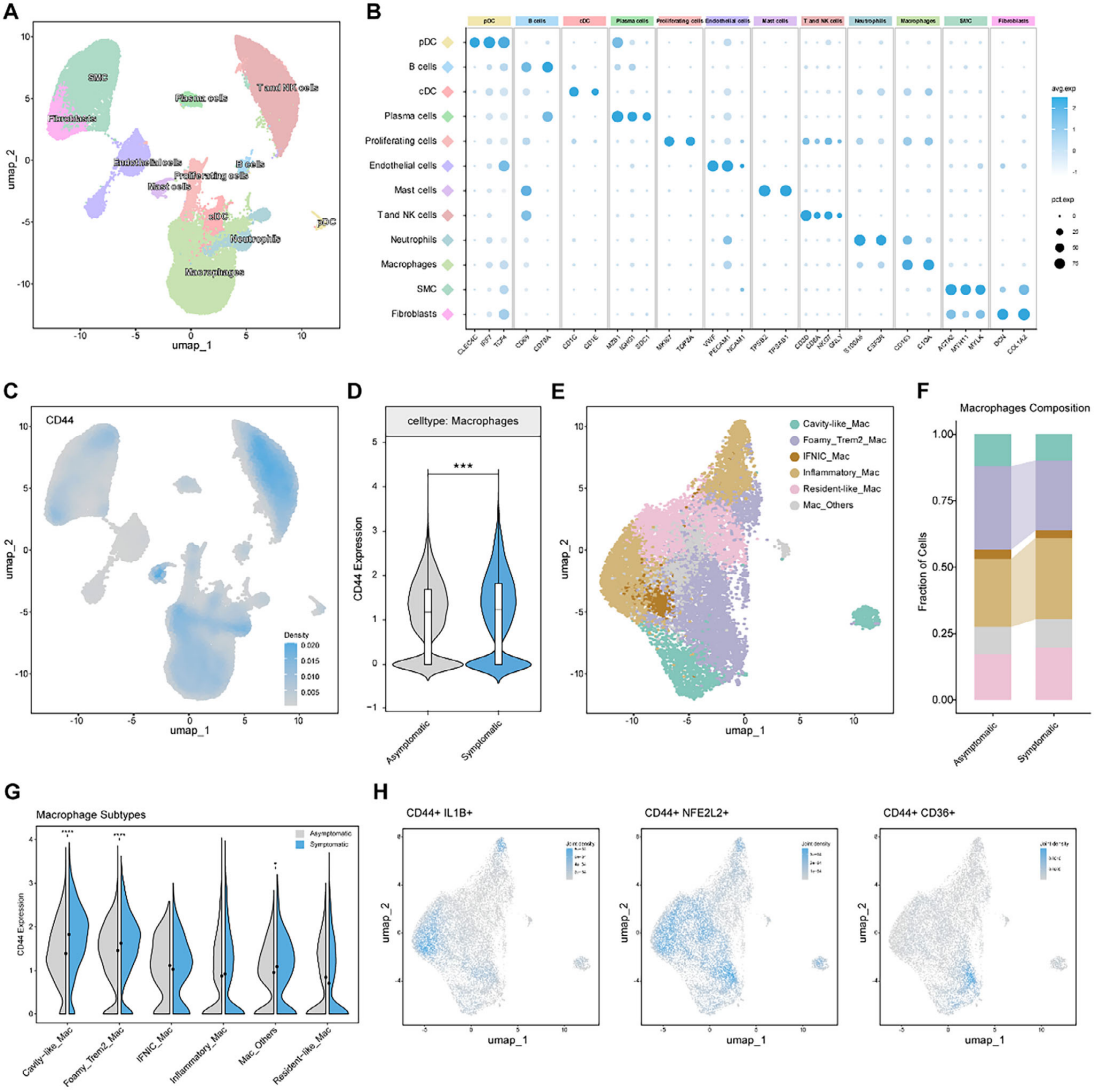
Single‐cell transcriptomics identifies CD44 as a potential targeting receptor on pathogenic macrophages in atherosclerotic lesions. (A) UMAP projection of the human carotid plaque single‐cell transcriptomic dataset (GSE253903), illustrating the distinct clustering of major immune and stromal cell lineages. (B) Dot plot depicting the expression profiles of cell‐type‐specific marker genes across all identified clusters. (C) Density Plot showing the high expression of CD44. (D) Violin plots demonstrate significantly elevated CD44 expression in macrophages from symptomatic patients compared to asymptomatic patients. (E) UMAP sub‐clustering of the macrophage population into distinct functional subsets. (F) Bar graph showing an increased proportion of inflammatory macrophages and a decreased proportion of Foamy_Trem2 macrophages in symptomatic lesions. (G) Violin plots detailing the differential expression of CD44 across macrophage subtypes between the two clinical groups. (H) Density Plot illustrating the strong co‐expression of CD44 with pathogenic markers (IL1B, NFE2L2, and CD36).

To further dissect macrophage heterogeneity, we performed sub‐clustering analysis, identifying six functionally distinct macrophage subsets based on their transcriptional profiles (Figure 2E). The canonical and subtype‐specific marker genes used to define these annotated macrophage populations were detailed via transcriptional profiling (Figure S5). Compositional analysis of these subsets revealed a distinct phenotypic shift associated with disease severity; symptomatic lesions exhibited a marked expansion of the Inflammatory macrophage subset and a corresponding reduction in the Foamy_Trem2 subset (Figure 2F,G). Transcriptional profiling of classical functional markers corroborated this microenvironmental shift: within symptomatic macrophage sub‐clusters, pro‐inflammatory M1 markers (HLA‐DRA, IL1B) were significantly upregulated (Figure S6), while anti‐inflammatory or pro‐resolving M2 markers (CD163, MS4A4A) were concurrently downregulated (Figure S7). UMAP overlays demonstrated that cells with high CD44 expression strongly co‐localized with macrophage populations enriched for key pathogenic mediators, specifically IL1B, NFE2L2, and CD36 (Figure 2H). Therefore, the highly expressed CD44 within atherosclerotic plaques is closely associated with inflammatory responses, oxidative stress, and lipid metabolic dysregulation, supporting CD44 as a plaque‐associated target for subsequent targeted therapy of atherosclerosis.

### 2.2 Physicochemical Characterization and Multi‐Enzymatic Antioxidant Activities of CuPB@HA Nanozymes

To validate the successful synthesis and structural integrity of the CuPB@HA nanozymes, we conducted a comprehensive suite of physicochemical analyses. Transmission electron microscopy (TEM) and high‐resolution TEM revealed that CuPB@HA possesses a well‐defined, crystalline cubic morphology (Figure 3A,B). Energy‐dispersive X‐ray spectroscopy (EDS) and elemental mapping confirmed that CuPB@HA is composed of Cu, Fe, C, K, N, and O, which are homogeneously distributed throughout the nanostructure (Figure 3C,D). The formation of the PB framework was further supported by the characteristic cubic morphology, crystalline diffraction pattern, and Fe‐related XPS signals of PB nanoparticles, as shown in the Supporting Information (Figures S8A, S9A, and S10A).

**FIGURE 3:**
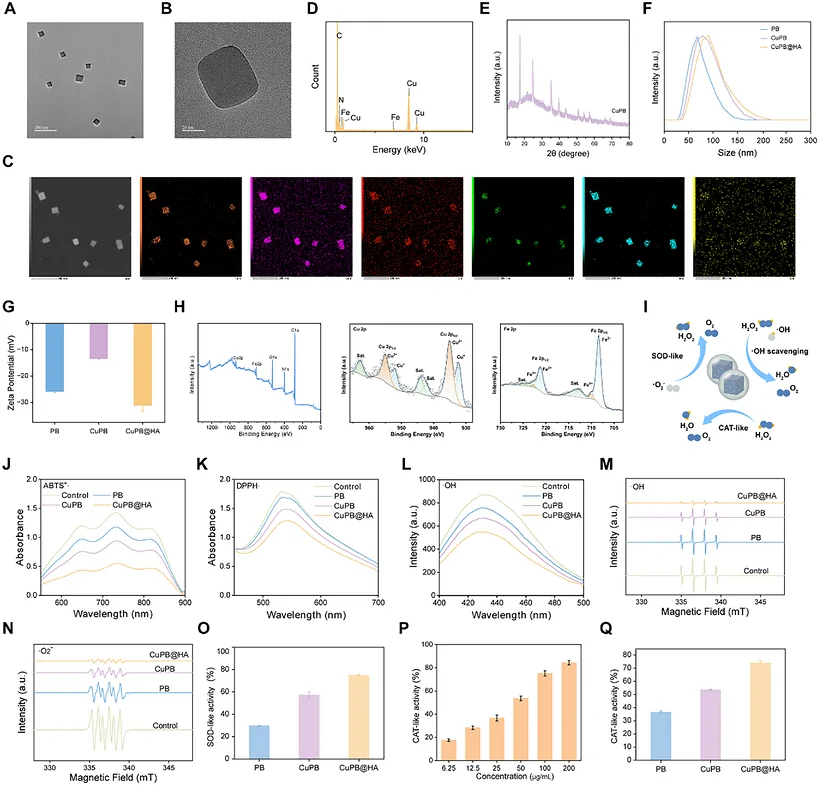
Physicochemical characterization and multi‐faceted enzyme‐mimicking antioxidant activities of the synthesized nanozymes. (A) TEM images of CuPB@HA (scale bars: 200 nm). (B) High‐resolution TEM image further illustrating the crystalline nature of CuPB@HA (scale bars: 20 nm). (C) Elemental mapping of CuPB@HA with Fe, Cu, C, K, N, and O. (D) EDS spectrum confirming the elemental composition of CuPB@HA. (E) XRD of CuPB. (F) Hydrodynamic size distributions of PB, CuPB, and CuPB@HA. (G) Zeta potential of PB, CuPB, and CuPB@HA. (H) XPS analysis of CuPB for elemental valence states. (I) Schematic illustration depicting the multifaceted enzyme‐mimicking and broad‐spectrum ROS‐scavenging mechanisms of the CuPB@HA nanozyme. The ability to scavenge free radicals of (J) ABTS^+^·, (K) DPPH· and (L‐M) ·OH. (N‐O) SOD‐like enzyme‐mimicking activity with PB, CuPB and CuPB@HA. (P) The CAT‐like function of CuPB@HA at various concentrations. (Q) The CAT‐like activity of CuPB@HA was compared with that of PB and CuPB.

Additionally, X‐ray diffraction (XRD) verified the distinct crystalline phases of the CuPB (Figure 3E). Moreover, Cu incorporation preserved the crystal phase of Prussian blue while leaving the particle size largely unchanged (Figure 3F), and TEM observation showed that the CuPB nanoparticles maintained a typical nanocubic morphology after copper incorporation (Figure S8B). After HA coating, CuPB@HA still retained the characteristic PB‐like diffraction peaks, indicating that HA functionalization did not disrupt the crystalline framework of the CuPB (Figure S9B). However, a pronounced decrease in zeta potential was observed after HA modification, indicating the successful coating of HA on the nanoparticle surface (Figure 3G). This surface modification was further verified by FT‐IR spectroscopy. CuPB@HA retained the characteristic C≡N stretching vibration of the PB‐based framework at approximately 2080–2100 cm^−^^1^, while showing additional HA‐associated absorption features in the fingerprint region, including carboxylate‐related vibrations around 1600–1650 and 1410–1450 cm^−^^1^ and polysaccharide backbone vibrations around 1050–1150 cm^−^^1^ (Figure S11). TGA further showed greater thermal weight loss and lower residual mass in CuPB@HA than in CuPB, indicating the presence of an additional organic HA component (Figure S12). Based on residual mass normalization at 800°C, the HA loading content in CuPB@HA was estimated to be approximately 26.1 wt.%. XPS analysis of CuPB revealed the coexistence of Cu^2^^+^ and Cu^+^, with a Cu^2^^+^/Cu^+^ ratio of 2.01, indicating the redox‐active nature of the CuPB catalytic core (Figure 3H). The Cu 2p XPS spectrum of CuPB@HA further confirmed that Cu‐related valence features were retained after HA coating (Figure S10B).

Building upon this structural foundation, we systematically evaluated the broad‐spectrum ROS‐scavenging capacity and multi‐enzymatic capabilities of CuPB@HA (Figure 3I). Quantitative in vitro assays highlighted the rapid and superior antioxidant capacity of the nanozymes, evidenced by significant ABTS (2,2′‐Azino‐bis(3‐ethylbenzothiazoline‐6‐sulfonic acid)) and DPPH (1,1‐Diphenyl‐2‐picrylhydrazyl) free radical scavenging activities (Figure 3J,K), with evident radical‐scavenging effects observed at 100 µg/mL (Figures S13 and S14), as well as a robust ability to quench highly reactive hydroxyl radicals (·OH) (Figures 3L,M and S15). Furthermore, copper doping significantly enhanced the multi‐enzymatic cascade efficiency of the nanozyme. CuPB@HA exhibited potent SOD‐like activity, driving the high‐efficiency decomposition of ·O_2_^−^ (Figures 3N,O and S16), alongside pronounced CAT‐like activity (Figure 3P,Q). Collectively, these findings indicate that Cu incorporation enhances the redox‐catalytic activity of the PB framework, enabling CuPB@HA to function as an efficient antioxidant nanozyme for mitigating complex oxidative stress microenvironments.

### 2.3 Targeted Plaque Accumulation and Synergistic Macrophage Remodeling by CuPB@HA

To evaluate the plaque‐targeting potential and cellular regulatory effects of CuPB@HA, we first investigated the in vivo distribution of Cy5.5‐labeled nanozymes in HFD‐fed ApoE^−/−^ atherosclerotic mice. Whole‐body fluorescence imaging showed dynamic systemic distribution of the nanozymes after intravenous administration, with detectable fluorescence signals at 12 h and 24 h post‐injection (Figure 4A). Ex vivo fluorescence imaging of major organs further showed fluorescence signals in the liver and lung, and CuPB@HA also exhibited detectable fluorescence in the heart region (Figure 4B). To further determine whether the accumulated nanozymes were associated with plaque macrophage‐rich regions, we performed confocal imaging of atherosclerotic plaque sections from HFD‐fed ApoE^−/−^ mice. The results showed that Cy5.5‐labeled CuPB@HA signals partially overlapped with CD68‐positive macrophage‐rich areas and CD44‐positive regions within plaques, supporting the effective enrichment of CuPB@HA in CD44‐associated macrophage‐rich plaque microenvironments (Figure 4C).

**FIGURE 4:**
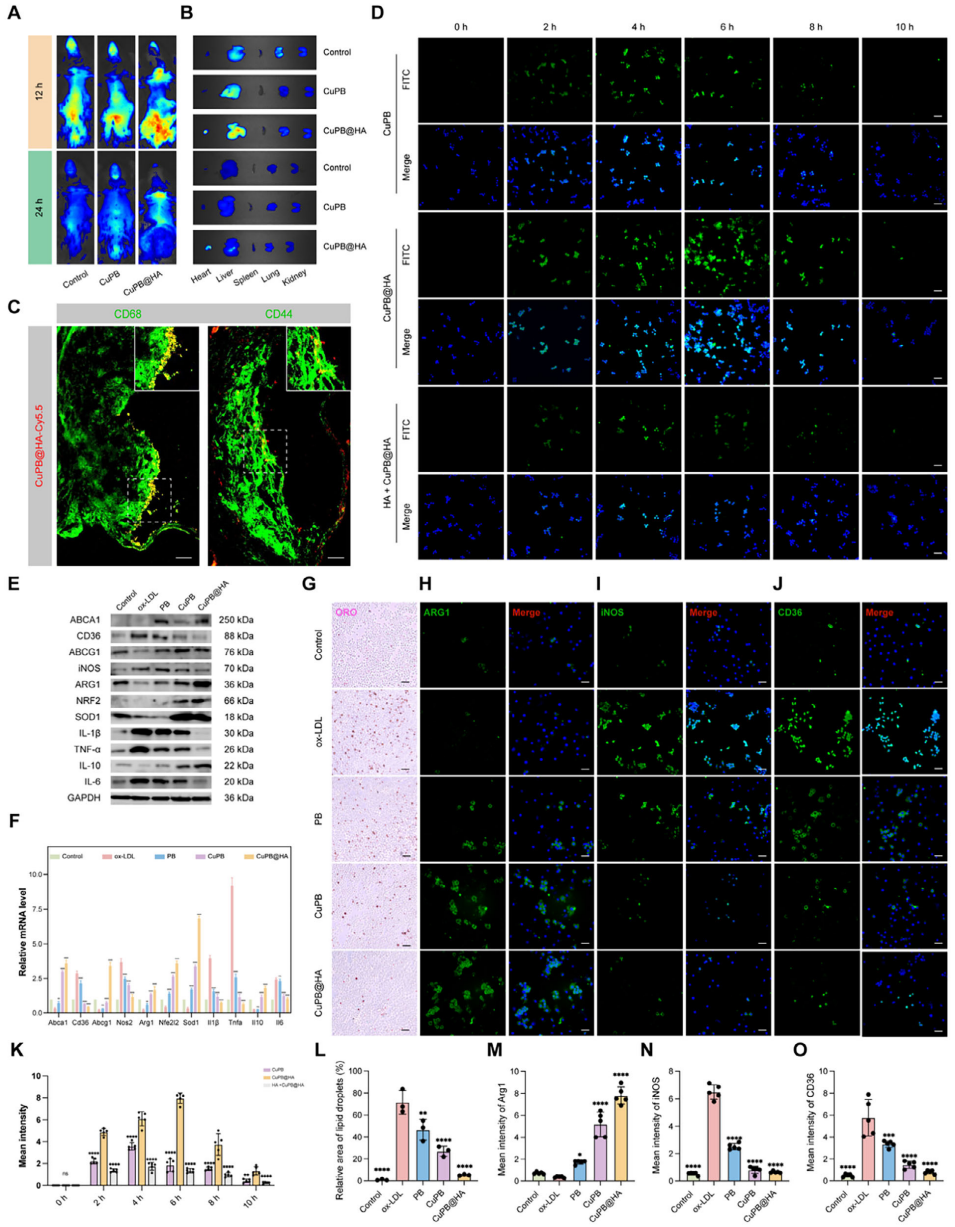
CuPB@HA nanozymes accumulate in atherosclerotic plaques and synergistically remodel lipid metabolism, oxidative stress, and inflammatory polarization in macrophages. (A) Representative in vivo fluorescence images of HFD‐fed ApoE^−/−^ atherosclerotic mice after intravenous administration of Cy5.5‐labeled CuPB or CuPB@HA at 12 and 24 h post‐injection. (B) Ex vivo fluorescence images of major organs, including heart, liver, spleen, lung, and kidney, harvested at corresponding time points after nanozyme administration. (C) Representative confocal fluorescence images of atherosclerotic plaque sections from HFD‐fed ApoE^−/−^ mice showing the spatial association of Cy5.5‐labeled CuPB@HA with CD68‐positive macrophage‐rich regions and CD44‐positive regions. Cy5.5‐labeled CuPB@HA is pseudo‐colored red, CD68 or CD44 is shown in green. (D) Fluorescence microscopy images showing the time‐dependent cellular uptake of FITC‐labeled CuPB and CuPB@HA by macrophages, with or without excess free HA pre‐incubation. FITC‐labeled nanozymes are shown in green, and nuclei are stained with DAPI in blue. (E) Western blot analysis of proteins related to lipid metabolism, oxidative stress, and inflammatory polarization in RAW264.7 macrophages after different treatments. (F) RT‐qPCR analysis of genes related to lipid metabolism, oxidative stress, and inflammatory polarization in RAW264.7 macrophages after different treatments. (G) Representative Oil Red O staining images showing intracellular lipid accumulation in RAW264.7 macrophages after different treatments. (H–J) Representative immunofluorescence images showing the expression of ARG1 (H), iNOS (I), and CD36 (J) in RAW264.7 macrophages after different treatments. (K) Quantitative analysis of cellular uptake fluorescence intensity in Figure 4D. (L) Quantitative analysis of Oil Red O‐positive areas in Figure 4G (n = 3). (M–O) Quantitative fluorescence analysis of ARG1 (M), iNOS (N), and CD36 (O) staining in Figure 4H–J (*n* = 5). Quantitative data are presented as the mean ± SD. Statistical significance was assessed via one‐way ANOVA (*p < 0.05, ** *p* < 0.01, *** *p* < 0.001, **** *p* < 0.0001).

Before evaluating cellular internalization, we first assessed the cytocompatibility of the nanozymes. CCK‐8 assays confirmed that both CuPB and CuPB@HA showed good cytocompatibility in RAW264.7 macrophages and THP‐1‐derived macrophage‐like cells within the tested concentration range of 0–200 µg/mL (Figure S17). Combined with the antioxidant activity observed at 100 µg/mL, this concentration was used for subsequent cellular uptake and functional assays. Fluorescence microscopy using FITC‐labeled nanozymes showed time‐dependent macrophage uptake, with CuPB@HA displaying stronger intracellular fluorescence than CuPB at the same time points. Moreover, pre‐incubation with excess free HA attenuated CuPB@HA uptake, supporting the involvement of HA‐mediated receptor interactions in the cellular internalization process (Figure 4D).

To further dissect the in vitro anti‐atherosclerotic efficacy of CuPB@HA, we systematically evaluated its regulatory effects on lipid metabolism, oxidative stress, and inflammatory polarization. Western blot and RT‐qPCR analyses showed that CuPB@HA dynamically remodeled ox‐LDL‐induced macrophage dysfunction in RAW264.7 macrophages (Figures 4E,F and S18). Consistently, THP‐1 macrophages cells also exhibited similar regulatory responses after CuPB@HA treatment (Figure S19). Specifically, CuPB@HA downregulated the lipid uptake receptor CD36 while upregulating the cholesterol efflux‐related transporters ABCA1 and ABCG1, suggesting an improved lipid‐handling capacity. Meanwhile, CuPB@HA enhanced antioxidant defense‐related markers, including NRF2 and SOD1, and shifted macrophages away from a pro‐inflammatory phenotype, as reflected by decreased iNOS, IL‐1β, TNF‐α, and IL‐6 levels together with increased ARG1 and IL‐10 levels.

This lipid‐regulatory effect was visually corroborated by Oil Red O staining, which demonstrated that nanozyme treatment suppressed intracellular lipid accumulation (Figure 4G). Quantitative analysis of Oil Red O‐positive areas further confirmed the reduction in lipid deposition after CuPB@HA treatment (Figure 4L). In addition, concentration‐gradient Oil Red O staining showed that CuPB@HA suppressed intracellular lipid accumulation in a concentration‐dependent manner, with the inhibitory effect becoming more pronounced at higher concentrations and approaching a plateau within the 100–200 µg/mL range (Figure S20). These phenotypic changes were further visualized by immunofluorescence staining, which showed enhanced ARG1 expression, reduced iNOS expression, and decreased CD36 expression after CuPB@HA treatment (Figure 4H–J). Quantitative fluorescence analysis further confirmed these trends (Figure 4M–O). Together with the in vivo plaque accumulation and cellular uptake results, these findings indicate that CuPB@HA effectively accumulates in CD44‐associated macrophage‐rich plaque microenvironments and synergistically regulates lipid metabolism, oxidative stress, and inflammatory polarization in macrophages.

### 2.4 CuPB@HA Nanozymes Restore Lipid Metabolic Balance and Promote Macrophage Phenotypic Remodeling

To elucidate the molecular mechanisms underlying the therapeutic efficacy of CuPB@HA nanozymes, we performed bulk RNA‐sequencing (RNA‐seq) on macrophage foam cells. Following data normalization (Figure S21), principal component analysis (PCA) demonstrated distinct clustering and minimal intra‐group variance among the experimental cohorts (Figure S22). Differential expression analysis revealed that ox‐LDL stimulation induced widespread transcriptomic alterations, identifying 568 differentially expressed genes (DEGs) (180 upregulated and 388 downregulated) in the Model group compared to the Control. Focusing on the upregulated DEGs (Figure 5A), Gene Ontology (GO) enrichment analysis indicated that the disease model predominantly activated biological processes associated with inflammatory cascades (e.g., NF‐kB and interleukin signaling) and lipid metabolic reprogramming, including lipid localization and transport (Figure 5B). Conversely, CuPB@HA intervention substantially reversed this pathogenic transcriptional profile. Comparison between the Treat (CuPB@HA) and Model groups yielded 549 DEGs (350 upregulated and 199 downregulated). GO enrichment analysis of the downregulated DEGs (Figure 5C) demonstrated that CuPB@HA effectively suppressed the pro‐atherosclerotic pathways activated during foam cell formation, particularly mitigating the inflammatory response and macrophage‐derived foam cell differentiation processes (Figure 5D).

**FIGURE 5:**
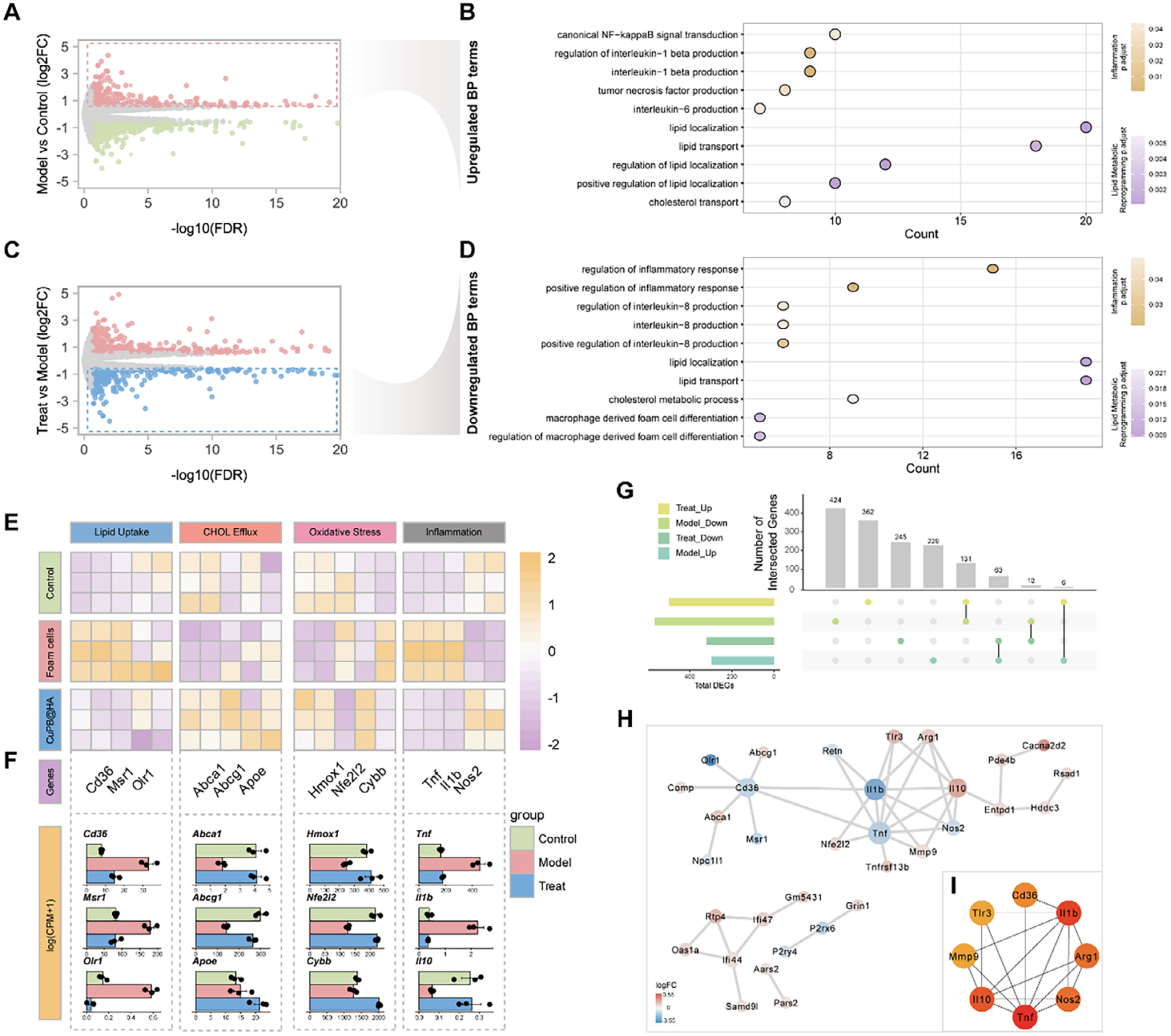
Transcriptomic reprogramming of pathogenic macrophages by CuPB@HA nanozymes. (A) Differential expression scatter plot of Model vs. Control, highlighting upregulated DEGs (red, Fold Change > 1.5, *FDR* < 0.05). (B) GO biological process enrichment of the upregulated DEGs from (A). (C) Differential expression scatter plot of Treat vs. Model, highlighting downregulated DEGs (blue, Fold Change > 1.5, FDR < 0.05). (D) GO biological process enrichment of the downregulated DEGs from (C). (E) Heatmap of representative DEGs for lipid uptake, cholesterol efflux, oxidative stress, and inflammation. (F) Quantitative expression profiles of essential genes selected from (E). Data are mean ± SD (*n* = 3). (G) UpSet plot showing the intersection of DEGs between the disease progression and treatment sets. (H) Protein‐protein interaction (PPI) network of the key intersected DEGs. (I) Core PPI sub‐network of highly interconnected hub genes (Cd36, Il1b, Tnf, Il10, Nos2, Arg1, Mmp9).

To further delineate the regulation of anti‐atherosclerotic functional modules, we visualized the expression patterns of specific genes directly involved in inflammation, oxidative stress, and lipid metabolism. Heatmaps visually confirmed the overall restoration of these functional signatures within the treatment group (Figure 5E). Quantitative extraction of specific transcript levels verified this trend: CuPB@HA significantly suppressed the expression of key lipid uptake receptors (Cd36, Msr1, Olr1) and pro‐inflammatory cytokines (Tnf, Il1b, Nos2), while substantially partially restoring the expression of cholesterol efflux mediators (Abca1, Abcg1) and antioxidant/anti‐inflammatory factors (Hmox1, Nfe2l2, Il10) (Figure 5F). Additionally, we evaluated other lipid metabolism‐related modules, including cholesterol esterification, lipid droplet dynamics, fatty acid synthesis, fatty acid oxidation, and fatty acid transport. Transcriptional profiling of these specific subsets revealed that CuPB@HA treatment effectively reversed various transcriptional dysregulations associated with the macrophage‐to‐foam cell transition (Figure S23). To pinpoint the core molecular targets mediating this transcriptomic reprogramming, we intersected the disease‐induced and treatment‐reversed transcriptomes. By filtering for genes that were upregulated in the Model but subsequently downregulated by treatment, as well as those downregulated in the Model but rescued by treatment, we isolated a highly specific subset of 194 functionally reversed DEGs (Figure 5G). Utilizing a high‐confidence protein‐protein interaction (PPI) threshold (score > 0.7), these 194 genes were mapped to reveal a densely interconnected molecular network governing inflammation and lipid regulation (Figure 5H). Extraction of the core functional module from this network highlighted a central regulatory hub featuring key DEGs such as Cd36, Il1b, Tnf, Arg1, and Nos2, identifying them as the primary molecular targets acted upon by CuPB@HA to synergistically rescue the macrophage phenotype (Figure 5I). Collectively, these transcriptomic findings provide mechanistic evidence that CuPB@HA alleviates macrophage dysfunction by coordinately regulating lipid metabolism, inflammatory activation, and redox homeostasis.

### 2.5 CuPB@HA Attenuates Atherosclerotic Burden and Promotes Plaque Stability‐Associated Features In Vivo

To translate our in vitro findings into a physiological context, we evaluated the anti‐atherosclerotic efficacy of CuPB@HA using an ApoE^−/−^ mouse model fed a high‐fat diet (HFD). Eight‐week‐old mice were maintained on the HFD for a total of 12 weeks. Targeted therapeutic intervention was initiated three weeks prior to the termination of the HFD regimen, with intravenous injections administered twice a week for a total of five doses (Figure 6A). In the in vivo experiments, the Control group refers to age‐matched wild‐type C57BL/6 mice, whereas the Saline group refers to HFD‐fed ApoE^−/−^ mice treated with saline. Serum lipid analysis showed that HFD‐fed ApoE^−/−^ mice exhibited marked dyslipidemia, as reflected by increased total cholesterol (TC), triglycerides (TG), and low‐density lipoprotein cholesterol (LDL‐C), together with decreased high‐density lipoprotein cholesterol (HDL‐C). Compared with the Saline group, CuPB@HA treatment reduced serum TC, TG, and LDL‐C levels by approximately 21.2%, 30.0%, and 22.8%, respectively, while increasing HDL‐C levels by approximately 20.3%, indicating an improvement in systemic lipid profiles under the tested conditions (Figure 6B–E). Macroscopic evaluation of the entire aorta by en face Oil Red O (ORO) staining showed that CuPB@HA treatment reduced aortic lipid lesion area compared with the Saline group (Figure 6K). Consistently, ORO staining of aortic root cross‐sections further demonstrated that CuPB@HA reduced intraplaque lipid deposition by approximately 67.0% compared with the Saline group (Figure 6L).

**FIGURE 6:**
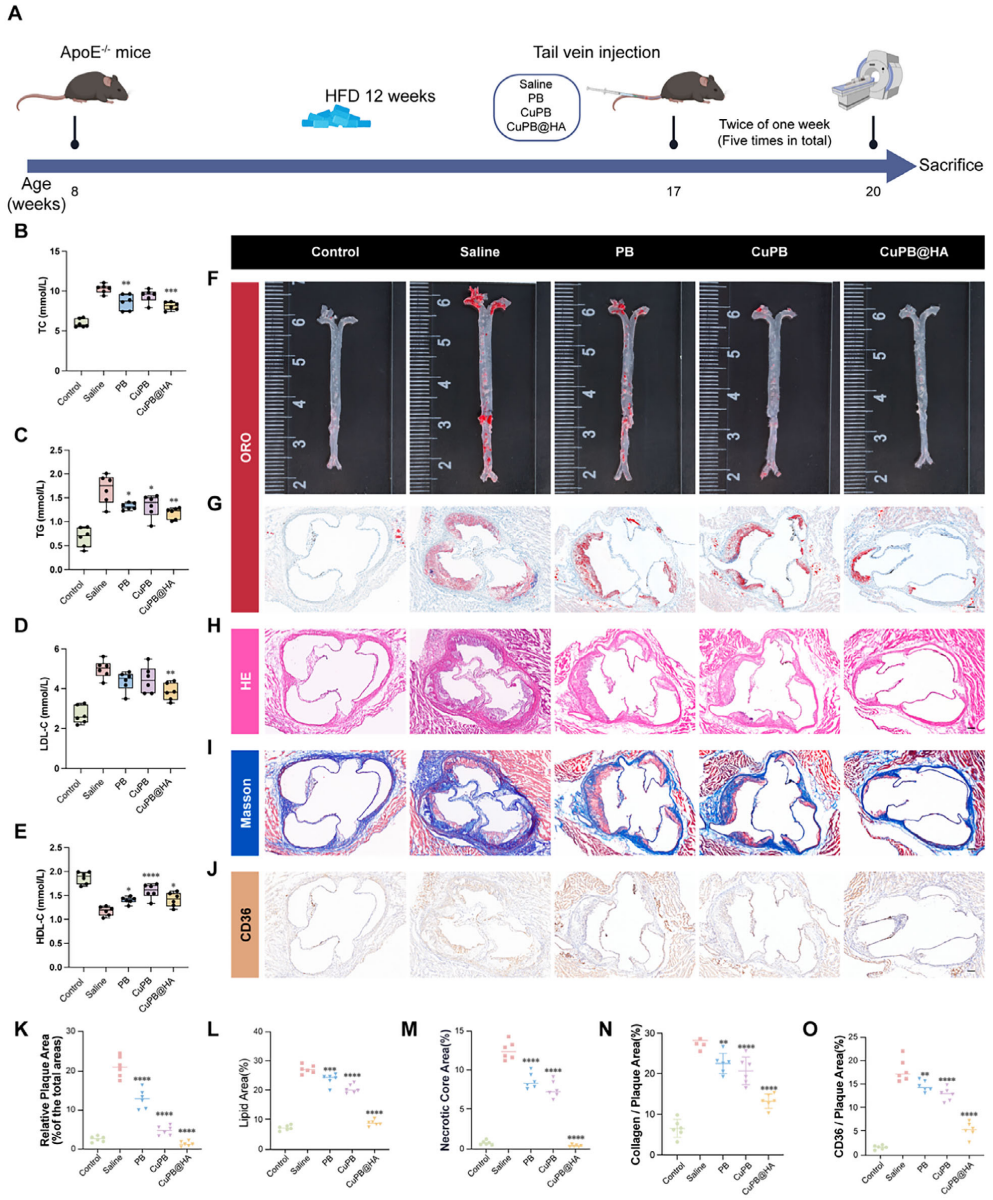
CuPB@HA attenuates atherosclerotic plaque burden and promotes plaque stability in HFD‐fed ApoE^−/−^ mice. (A) Schematic of the in vivo experimental design and treatment timeline. (B–E) Serum lipid profiles of mice in different treatment groups, including (B) total cholesterol (TC), (C) triglycerides (TG), (D) low‐density lipoprotein cholesterol (LDL‐C), and (E) high‐density lipoprotein cholesterol (HDL‐C). Data are mean ± SD (*n* = 6). Significance was assessed via one‐way ANOVA with Tukey's post hoc test (*p < 0.05, ** *p* < 0.01, *** *p* < 0.001, **** *p* < 0.0001). (F–J) Representative histological and immunohistochemical images of aortic root cross‐sections (scale bars: 100 µm): (F) Representative Oil Red O (ORO) staining of aortas. (G) ORO staining for lipid accumulation; (H) H&E staining for necrotic core and plaque morphology; (I) Masson's trichrome staining for collagen deposition; and (J) IHC staining for CD36 expression. (K–O) Quantification of lesional characteristics across treatment groups: (K) relative plaque area (*en face* ORO), (L) lipid area (aortic root ORO), (M) necrotic core area (H&E), (N) collagen‐to‐plaque ratio (Masson's trichrome), and (O) CD36‐positive area (IHC). Data are presented as the mean ± SD (*n* = 6). Significance was assessed via one‐way ANOVA with Tukey's post hoc test (*p < 0.05, ** *p* < 0.01, *** *p* < 0.001, **** *p* < 0.0001).

We next evaluated plaque morphology and stability‐associated histological features. Hematoxylin and eosin (H&E) staining showed that CuPB@HA treatment reduced necrotic core area within aortic root plaques (Figure 6H,M). Masson's trichrome staining revealed increased collagen deposition in plaques after CuPB@HA treatment, suggesting improvement of plaque stability‐associated extracellular matrix features (Figure 6I,N). In addition, immunohistochemical staining showed that CuPB@HA reduced the CD36‐positive plaque area by approximately 70.0% compared with the Saline group, consistent with decreased lipid uptake‐associated signaling within the plaque microenvironment (Figure 6J,O). To further assess plaque stability‐associated features, we performed immunofluorescence staining for matrix metalloproteinase‐9 (MMP9) and α‐smooth muscle actin (α‐SMA) in aortic root sections. Compared with the Saline group, CuPB@HA treatment decreased MMP9 fluorescence intensity and increased α‐SMA fluorescence intensity (Figure S24), supporting reduced matrix‐degrading activity and increased smooth muscle‐related fibrous structural features within the plaques.

Together, these in vivo results indicate that CuPB@HA attenuates plaque burden, improves systemic lipid profiles, and promotes plaque stability‐associated histological features in HFD‐fed ApoE^−/−^ mice.

### 2.6 In Vivo Biodistribution, Lesional Targeting, and Systemic Biosafety Profile of CuPB@HA

To non‐invasively evaluate the therapeutic efficacy, we performed magnetic resonance imaging (MRI) of the carotid arteries on the final day of treatment, using a uMR 9.4 T MRI system (United Imaging Life Science Instrument, China) before sample collecting for histological staining. The specific anatomical region of the carotid arteries subjected to scanning is detailed in Figure S25. The acquired images and their corresponding high‐magnification cross‐sections clearly delineated the plaque morphology, visually confirming that targeted CuPB@HA intervention significantly alleviated plaque‐induced vessel wall thickening (Figure 7A).

**FIGURE 7:**
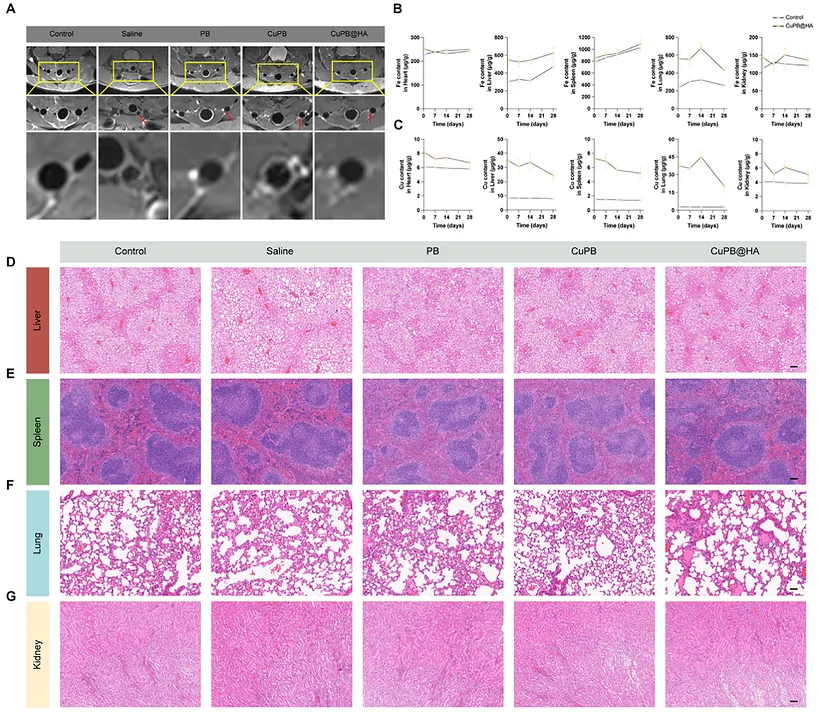
In vivo plaque imaging, quantitative biodistribution, and systemic biosafety evaluation of CuPB@HA nanozymes. (A) In vivo MRI of the carotid arteries in different treatment groups (*n* = 3). (B, C) Time‐dependent quantitative biodistribution of Fe (B) and Cu (C) in major organs, including heart, liver, spleen, lung, and kidney, after intravenous administration of CuPB@HA in C57BL/6 mice. Age‐matched C57BL/6 mice injected with an equivalent volume of saline at each corresponding time point were used to determine endogenous Fe and Cu background levels. Elemental contents were measured by ICP analysis at 1, 7, 14, 21, and 28 days post‐injection (*n* = 3). (D–G) Representative Hematoxylin and Eosin (H&E) stained histological sections of the major organs (scale bars: 100 µm), including (D) liver, (E) spleen, (F) lung, and (G) kidney, from the Control, Saline, PB, CuPB, and CuPB@HA groups.

To further evaluate the in vivo biodistribution and clearance behavior of CuPB@HA, we performed an independent time‐course ICP analysis in C57BL/6 mice. After intravenous administration of CuPB@HA, major organs, including the heart, liver, spleen, lung, and kidney, were harvested at 1, 7, 14, 21, and 28 days post‐injection for Fe and Cu quantification. Age‐matched C57BL/6 mice injected with an equivalent volume of saline at each corresponding time point were used to determine endogenous Fe and Cu background levels. The Fe contents exhibited organ‐dependent dynamic changes over time. Compared with the saline controls, CuPB@HA‐treated mice generally showed elevated Fe levels in several organs, indicating in vivo deposition and subsequent processing of the Prussian Blue framework. Among the examined organs, the liver and lung displayed more evident Fe variations, consistent with their involvement in nanomaterial distribution and clearance, whereas the heart and kidney showed comparatively modest changes (Figure 7B). In parallel, endogenous Cu levels in saline‐treated mice remained relatively low and stable over time, reflecting physiological copper homeostasis. After CuPB@HA administration, Cu contents increased in all examined organs to varying extents, with more pronounced signals observed in the liver and lung, while the heart and kidney showed relatively limited changes. Notably, after the initial elevation, Cu levels in several organs gradually decreased during the later observation period, particularly from 21 to 28 days, suggesting a time‐dependent clearance trend of CuPB‐derived copper in vivo (Figure 7C).

Finally, to evaluate the systemic biocompatibility of CuPB@HA under the tested conditions, we monitored the body weight of mice throughout the in vivo study. No animal in any group exhibited body weight loss exceeding 15% of its initial body weight at any time point (Figure S26). In addition, liver function indicators (Figure S27), kidney function indicators (Figure S28), and hematological parameters (Figure S29) showed no obvious treatment‐related abnormalities among the experimental groups. We also conducted histopathological examinations of major organs, including the liver (Figure 7D), spleen (Figure 7E), lung (Figure 7F), and kidney (Figure 7G), using H&E staining. Microscopic observations revealed no obvious pathological alterations or tissue necrosis across the treatment cohorts, supporting the short‐term in vivo biocompatibility of the engineered nanozymes under the tested conditions.

## 3 Discussion

In this study, we developed hyaluronic acid‐functionalized copper‐doped Prussian blue nanozymes (CuPB@HA) for CD44‐associated plaque targeting. CuPB@HA preferentially accumulated in CD44‐positive macrophage‐rich plaque regions and helped attenuate the maladaptive cycle of oxidative stress, lipid accumulation, and inflammatory activation. Upon internalization, CuPB@HA exhibited multi‐enzymatic ROS‐scavenging activity, which was associated with reduced CD36 expression, increased ABCA1/ABCG1 expression, improved macrophage cholesterol‐handling mediator profiles, and a shift toward an anti‐inflammatory macrophage phenotype. In vivo, CuPB@HA reduced plaque burden and improved plaque stability‐associated histological features in HFD‐fed ApoE^−/−^ mice.

The rationale for CD44‐associated plaque targeting is supported by recent advances in single‐cell RNA sequencing (scRNA‐seq) and spatial transcriptomics, which have revealed profound heterogeneity among lesional immune and stromal cells [21]. In our analysis of human carotid plaque scRNA‐seq data, CD44 was broadly expressed across plaque‐associated cell populations, with evident signals in macrophage populations. CD44‐expressing macrophages overlapped with inflammatory, oxidative‐stress‐related, and lipid‐metabolism‐related transcriptional programs, suggesting their relevance to the pathogenic plaque microenvironment. This observation was further supported by increased CD44 expression in aortic root plaques from HFD‐fed ApoE^−/−^ mice and in ox‐LDL‐induced RAW264.7 foam cells. These findings provided the rationale for using HA‐CD44 interactions to promote plaque‐associated localization of CuPB@HA. By employing HA as a biomimetic homing ligand, CuPB@HA links transcriptomic target discovery to CD44‐associated plaque delivery [22, 23, 24]. This strategy is consistent with recent advances in HA‐guided nanotherapeutics [25]. More broadly, CuPB@HA also fits within the emerging landscape of precision drug delivery, plaque‐directed nanomedicine, and immune‐targeted therapies for atherosclerosis [26].

Beyond plaque‐associated delivery, the therapeutic benefit of CuPB@HA is closely linked to its catalytic redox‐regulating capacity. Oxidative stress, inflammation, metabolic disturbance, and epigenetic dysregulation are interconnected drivers of atherosclerosis progression, particularly under diabetes‐associated vascular stress [27]. Within the atheroma, ROS continuously generated by metabolically stressed macrophages function as critical secondary messengers that sustain pathological polarization via the hyperactivation of HIF‐1α and NF‐kB signaling cascades [28, 29], closely mirroring recently identified metabolic vulnerabilities in atherogenic macrophages [30]. While traditional small‐molecule antioxidants often fail clinically due to rapid systemic clearance and poor intracellular bioavailability [31], transition metal‐doped nanozymes act as artificial, self‐renewing organelles [24, 32], a concept gaining significant traction in advanced catalytic nanomedicine [33].

In the present CuPB@HA system, the enhanced catalytic performance after Cu incorporation can be attributed to both electronic and biological factors. Prussian blue is an intrinsically redox‐active framework composed of mixed‐valence Fe centers, which provide catalytic sites for ROS conversion. The introduction of Cu further enriches the redox‐active centers within the PB lattice [34]. In particular, the reversible Cu^2^^+^/Cu^+^ redox cycling may facilitate electron transfer during ROS conversion, thereby accelerating superoxide dismutation and H_2_O_2_ decomposition. This additional Cu‐mediated redox pathway is consistent with our XPS results showing mixed Cu valence states in CuPB and with the enhanced SOD‐like and CAT‐like activities observed in CuPB@HA compared with PB [35, 36]. Beyond catalytic enhancement, Cu is an essential trace element involved in multiple biological processes, including mitochondrial metabolism, redox homeostasis, and ROS detoxification [37]. Therefore, Cu incorporation may improve the redox‐catalytic activity of the PB framework and contribute to redox regulation within the oxidative and inflammatory macrophage microenvironment.

To further explore potential signaling intermediates connecting ROS scavenging with macrophage cholesterol‐handling regulation, we performed a preliminary validation experiment using the LXR antagonist GSK2033 in ox‐LDL‐stimulated RAW264.7 macrophages. CuPB@HA restored the expression of LXRα, PPARγ, and ABCA1, while reducing CD36 expression. GSK2033 partially interfered with the CuPB@HA‐mediated regulation of LXRα‐ and ABCA1‐related responses, suggesting that LXR‐related signaling may participate in CuPB@HA‐mediated regulation of macrophage cholesterol‐handling mediators (Figure S30). However, because the effects on PPARγ and CD36 were not fully abolished, additional LXR‐independent mechanisms may also contribute. Thus, this experiment provides preliminary mechanistic support, and the precise transcriptional or epigenetic mechanisms linking transition‐metal‐mediated redox catalysis to lipid‐metabolism regulation remain to be further investigated using transcription factor activity assays, loss‐of‐function approaches, and epigenomic profiling.

Despite the therapeutic potential demonstrated by the CuPB@HA nanozyme platform, several limitations warrant further investigation before clinical translation can be realized. First, while the HFD‐fed ApoE^−/−^ mouse model is widely used in atherosclerosis research, murine lipid metabolism profiles, hemodynamic forces, and plaque architectures differ considerably from those in human patients. In addition, only male ApoE^−/−^ mice were used in this study to reduce sex‐related biological variability and maintain consistency with the preliminary experimental design. However, sex‐dependent differences in lesion development and plaque phenotype have been reported in ApoE^−/−^ mice, with several studies showing that female ApoE^−/−^ mice may develop larger or more advanced lesions under certain experimental conditions [38, 39, 40]. Therefore, future studies including both male and female mice are required to determine whether CuPB@HA exhibits sex‐specific biodistribution, therapeutic efficacy, or safety profiles. Moreover, the long‐term efficacy and safety of CuPB@HA should be further validated in large animal models, such as non‐human primates or hypercholesterolemic mini‐pigs, which better recapitulate human cardiovascular pathophysiology [41]. Second, although our short‐term systemic biosafety profiling and time‐course ICP analysis indicated no obvious acute tissue toxicity and suggested a time‐dependent clearance trend of CuPB@HA‐derived Fe/Cu signals in major organs, longer‐term pharmacokinetics, degradation dynamics, excretion routes, and potential delayed metal accumulation in the reticuloendothelial system still require further investigation. Recent studies on PB‐based and multifunctional nanozymes for atherosclerosis have shown that plaque inflammation can be modulated through integrated regulation of ROS scavenging, inflammatory resolution, macrophage behavior, lipid handling, and plaque microenvironment remodeling [42, 43]. These studies provide useful comparator frameworks for evaluating the design logic and biological effects of CuPB@HA. Nevertheless, the present study does not fully distinguish the independent contributions of HA coating, Cu incorporation, and possible copper ion release. Future studies should include HA‐only, HA‐coated PB, and CuCl_2_‐equivalent controls, as well as serum stability, protein‐corona analysis, endotoxin testing, and Fe/Cu leaching assays under physiological and lysosome‐mimicking acidic pH conditions, to further clarify the structural basis, biological interface, and biosafety profile of CuPB@HA. Addressing these limitations will help refine the design, safety evaluation, and translational potential of CuPB@HA‐based nanozyme therapy for atherosclerosis.

## 4 Conclusions

The targeted delivery of CuPB@HA nanozymes represents a promising preclinical strategy for atherosclerosis intervention. Through HA‐mediated CD44‐associated plaque targeting, this biomimetic platform preferentially accumulated in CD44‐positive macrophage‐rich plaque regions and contributed to the modulation of the pathogenic plaque microenvironment. The integration of copper‐enhanced ROS‐scavenging activity with macrophage lipid‐ and inflammation‐related regulation helped attenuate oxidative stress, reduce foam‐cell lipid accumulation, and suppress inflammatory activation. In particular, CuPB@HA decreased CD36 expression and increased the expression of cholesterol‐efflux‐related transporters ABCA1 and ABCG1, suggesting an improvement in macrophage cholesterol‐handling mediator profiles rather than direct proof of functional cholesterol efflux. In vivo, CuPB@HA reduced plaque burden, decreased necrotic core expansion, increased collagen deposition, reduced MMP‐9 expression, and increased α‐SMA‐positive s cell content, supporting partial improvement of plaque stability‐associated histological features.

Moving forward, although these preclinical findings support the therapeutic potential of CuPB@HA nanozymes, further studies are required before clinical translation. Future investigations should systematically evaluate long‐term pharmacokinetics, degradation, clearance, Fe/Cu leaching, protein‐corona formation, and systemic biosafety in larger animal models. Additional functional studies, including cholesterol efflux assays, ox‐LDL uptake assays, and loss‐of‐function validation of key lipid transporters, will also be needed to further clarify the mechanisms by which CuPB@HA regulates macrophage lipid handling and plaque inflammation.

## 5 Methods

### 5.1 Preparation And Optimization of PB, CuPB, and CuPB@HA Nanoparticles

The PB nanozymes were synthesized via a co‐precipitation method. Briefly, 40 mL of distilled water was mixed with 4 g polyvinylpyrrolidone (PVP, K30, Mw ∼ 50,000, Aladdin Reagent, Shanghai, China) under continuous magnetic stirring until completely dissolved. Subsequently, 132 mg potassium ferricyanide (K_3_[Fe(CN)_6_], Aladdin Reagent, Shanghai, China) was added to the solution and stirred for 1 h to ensure complete dissolution. Next, 33 µL of concentrated hydrochloric acid (HCl, Keshi, Chengdu, China) was introduced, and the mixture was stirred for 15 min. Thereafter, the reaction mixture was transferred to an oven and incubated at 80°C for 24 h. After completion, the final products were collected and washed three times with deionized water and ethanol by centrifugation (18,000 rpm, 15 min) to obtain the PB nanozymes.

The CuPB nanozymes were prepared using a similar coprecipitation procedure. Initially, 40 mL of distilled water was mixed with 4 g PVP under magnetic stirring until fully dissolved. Then, 132 mg K_3_[Fe(CN)_6_] was added and stirred for 1 h to obtain a homogeneous solution. After that, 33 µL of concentrated HCl was added, followed by stirring for 15 min. Subsequently, 6.86 mg copper chloride (CuCl_2_) was added, and the mixture was stirred for an additional 1 h. Finally, the reaction mixture was incubated at 80°C for 24 h. After the reaction, the CuPB nanozymes were collected and washed three times with deionized water and ethanol by centrifugation (18 000 rpm, 15 min), yielding the final CuPB products.

For surface functionalization, hyaluronic acid (HA) was conjugated to CuPB nanoparticles via carbodiimide chemistry. HA (47 mg) was dissolved in 5 mL deionized water. Under vigorous magnetic stirring, an aqueous solution of EDC (1‐Ethyl‐3‐(3‐dimethylaminopropyl) carbodiimide) (15.4 mg) and NHS (N‐Hydroxysuccinimide) (9.6 mg) in 10 mL deionized water was added dropwise, and the mixture was allowed to react for 3 h to obtain activated HA. The activated HA was then added dropwise to 8 mL of the previously prepared CuPB aqueous dispersion, and the mixture was kept under vigorous stirring in the dark for 3 days. After completion, the mixture was collected and centrifuged at 15,000 rpm for approximately 30 min. The obtained precipitate was re‐dispersed in 40 mL deionized water, and the washing step was repeated three times. Finally, the purified CuPB@HA nanozymes were lyophilized and stored for subsequent experiments.

### 5.2 Characterization Of PB, CuPB, and CuPB@Ha Nanozymes

The microstructures and surface morphologies of CuPB@HA were visualized using a 200‐kV high‐resolution transmission electron microscope (HRTEM, FEI Tecnai G2 F20). Dynamic light scattering (DLS) and electrophoretic mobility measurements were carried out on a Zetasizer Nano ZS90 platform (Malvern Panalytical) to track the changes in hydrodynamic size and zeta potential before and after HA coating. The crystal structure of the nanozymes was assessed on a Rigaku SmartLab X‐ray diffractometer. To investigate the surface elemental composition and the successful doping of copper ions, XPS spectra were acquired employing a Thermo Fisher K‐Alpha XPS system. Additionally, the presence of characteristic chemical bonds confirming the HA functionalization was verified through FTIR spectra collected via a Thermo Scientific Nicolet iS50 FTIR spectrometer.

### 5.3 Free Radical Scavenging and Enzyme‐Like Activity Assays

The free radical scavenging activities and enzyme‐like catalytic activities of PB, CuPB, and CuPB@HA were evaluated using ABTS^+^·, DPPH, ·OH scavenging assays, electron spin resonance (ESR) spectroscopy, SOD‐like activity assay, and CAT‐like activity assay.

For the ABTS^+^· assay, the ABTS^+^· working solution was generated by reacting ABTS with potassium persulfate in the dark for 12 h. Briefly, 30 mg of ABTS was dissolved in 7 mL of deionized water, and 7 mg of potassium persulfate was dissolved in 10 mL of deionized water. Then, 500 µL of ABTS solution was mixed with 500 µL of potassium persulfate solution and kept in the dark for 12 h. The resulting solution was diluted to 30 mL before use. Nanozymes solutions at different concentrations were mixed with the ABTS^+^· working solution and incubated for 10 min, followed by absorbance measurement at 734 nm.

For the DPPH assay, DPPH was dissolved in absolute ethanol to prepare a 0.03 mg mL^−^^1^ working solution. Nanozymes solutions at different concentrations were mixed with the DPPH solution and incubated for 10 min in the dark, followed by absorbance measurement at 519 nm. Separately, Hydroxyl radical (·OH) scavenging capacity was evaluated using the methyl violet method. Briefly, methyl violet solution, FeSO_4_ solution, Nanozymes solutions at different concentrations, and H_2_O_2_ solution were sequentially added to acidic buffer. After reaction for 10 min, the absorbance at 582 nm was measured.

ESR spectroscopy was used to evaluate ·OH and superoxide anion (·O_2_^−^) scavenging using 5,5‐dimethyl‐1‐pyrroline N‐oxide (DMPO) as the spin‐trapping agent. For ·OH detection, ·OH radicals were generated using a Fenton reaction system containing Fe^2^^+^ and H_2_O_2_. Nanozymes solutions at different concentrations were added to the reaction system before ESR measurement. For ·O_2_^−^ detection, ·O_2_^−^ radicals were generated using a riboflavin/UV irradiation system. Briefly, riboflavin solution was mixed with DMPO and Nanozymes solutions at different concentrations, followed by UV irradiation to generate ·O_2_^−^ radicals. The mixture was immediately transferred into a capillary tube for ESR measurement.

SOD‐like activity was assessed using a riboflavin/UV irradiation‐mediated superoxide generation system based on the nitroblue tetrazolium (NBT) method. Briefly, superoxide anions were generated by UV irradiation of riboflavin solution and reacted with NBT to form blue formazan, which was monitored at 560 nm. Nanozymes solutions at different concentrations were added to the reaction system before UV irradiation, and the decrease in absorbance at 560 nm was used to evaluate SOD‐like activity. CAT‐like activity was evaluated by monitoring dissolved oxygen generation during H_2_O_2_ decomposition using a dissolved oxygen meter. Briefly, Nanozymes solutions at different concentrations were added to H_2_O_2_ solution prepared in pH 7.4 buffer, and the dissolved oxygen concentration was continuously recorded.

### 5.4 Cell Culture

The murine macrophage cell line RAW264.7 (RRID:CVCL_C6XG) and THeP‐1 (RRID:CVCL_0006) were obtained from the Cell Bank of the Chinese Academy of Sciences (Shanghai, China). RAW264.7 macrophages were cultured in complete DMEM supplemented with 10% FBS (Gibco, Australia), 1% Pen‐Strep (100X, Gibco, USA), and 1% Non‐Essential Amino Acids (100X, Gibco, USA), and maintained in a humidified incubator at 37°C with 5% CO2. Cells were passaged when reaching 80%–90% confluence and then stimulated with ox‐LDL (100 µg/mL) for 48 h to induce foam cell formation. In addition, THP‐1 cells were differentiated into macrophages by incubating with PMA (100 ng/mL) for 24 h in RPMI‐1640 medium (Gibco–Thermo Fisher Scientific, Waltham, MA, USA) supplemented with 10% FBS, 50 µM β‐mercaptoethanol, and 1% penicillin/streptomycin (Gibco) at 37°C, 5% CO2. After differentiation, the cells were exposed to ox‐LDL (50 µg/mL) for 48 h to mimic atherosclerosis in vitro.

### 5.5 Cell Proliferation

The in vitro cytocompatibility of the engineered nanozymes was evaluated using the Cell Counting Kit‐8 (CCK‐8) assay (Yeasen Biotechnology, Shanghai, China). RAW264.7 macrophages were seeded into 96‐well plates at a density of 5,000 cells per well (100 µL/well) and incubated overnight at 37°C. THP‐1 cells were treated with PMA (100 ng/mL) to induce differentiation into macrophages, and then plated into 96‐well plates at a density of 3 × 10^4 cells per well (100 µL/well), followed by overnight incubation at 37°C to allow for cell adhesion. The original medium was replaced with fresh medium containing different concentrations (0, 3.125, 6.25, 12.5, 25, 50, 100, and 200 µg/mL) of CuPB or CuPB@HA. After 24 h of co‐incubation, 10 µL of CCK‐8 solution was added to each well, and the plates were further incubated for 1 h at 37°C in the dark. Finally, the optical density (OD) was measured at 450 nm using a microplate reader to calculate relative cell viability.

### 5.6 Cellular Internalization and Confocal Microscopy

To evaluate the macrophage‐targeting capability and intracellular uptake kinetics of the engineered nanozymes, RAW264.7 macrophages were seeded into confocal imaging dishes (5,000 cells/dish) and incubated overnight at 37°C. After removing the original medium, cells were incubated with fresh culture medium containing FITC‐labeled CuPB or FITC‐labeled CuPB@HA nanozymes (100 µg/mL). For the HA blocking group, cells were pre‐incubated with hyaluronic acid (HA) at a final concentration of 12.5 µg/mL for 1 h at 37°C before adding FITC‐labeled CuPB@HA nanozymes (100 µg/mL). Uptake was monitored at 0, 2, 4, 6, 8, and 10 h. At each time point, the cells were gently washed three times with cold PBS to remove non‐internalized nanozymes, then fixed with 4% paraformaldehyde (PFA) for 15 min at room temperature. After fixation and additional PBS washes, nuclei were stained with DAPI for 10 min at room temperature in the dark. Fluorescence images were acquired using a confocal laser scanning microscope with standard excitation/emission settings for DAPI (blue) and FITC (green). Image processing and uptake quantification were performed using ImageJ.

### 5.7 Western Blot

Cultured RAW264.7 macrophages were lysed in RIPA buffer supplemented with phosphatase and protease inhibitors, followed by quantification of total protein concentrations using a BCA Protein Assay Kit. Each of the protein samples was subsequently mixed with loading buffer (5×) at 4:1 (v/v) and heated at 100°C for 10 min to denature the proteins. Denatured protein samples were separated by electrophoresis on an SDS‐PAGE gel (80 V) and subsequently transferred to a PVDF membrane (290 mA), with the transfer process conducted at low temperature. After that, the membrane was blocked with 5% skimmed milk in TBST buffer at room temperature for 1.5 h and incubated with primary antibodies overnight at 4°C. The next day, the membrane was washed 3 times with TBST buffer, and then incubated with horseradish peroxidase (HRP)‐conjugated secondary antibodies at room temperature for 1 h. Finally, the target protein bands were developed using ECL luminescent reagent and quantitatively analyzed using ImageJ software.

Dilution for the primary antibodies: Rabbit anti‐CD36 (MedChemExpress, HY‐P86458 1:1000, RRID: AB_3740514). Rabbit anti‐ABCA1 (Proteintech, 26564‐1‐AP, 1:500, RRID: AB_3085884). Rabbit anti‐ABCG1 (Proteintech, 13578‐1‐AP, 1:2000, RRID: AB_2220174). Rabbit anti‐iNOS (Proteintech, 22226‐1‐AP, 1:1000, RRID: AB_2879038). Rabbit anti‐ARG1 (Proteintech, 16001‐1‐AP, 1:5000, RRID: AB_2289842). Rabbit anti‐NRF2 (Proteintech, 16396‐1‐AP, 1:4000, RRID: AB_2782956). Rabbit anti‐IL‐1β (Proteintech, 16806‐1‐AP, 1:3000, RRID: AB_10646432). Rabbit anti‐TNF‐α (Proteintech, 17590‐1‐AP, 1:2000, RRID: AB_2271853). Rabbit anti‐IL‐6 (Proteintech, 21865‐1‐AP, 1:500, RRID: AB_11142677). Rabbit anti‐IL‐10 (Proteintech, 60269‐1‐Ig, 1:2500, RRID: AB_2881389). Rabbit anti‐SOD1 (Proteintech, 10269‐1‐AP, 1:5000, RRID: AB_2193750). Rabbit anti‐GAPDH (Proteintech, 10494‐1‐AP, 1:8000, RRID: AB_2263076). Rabbit anti‐β‐actin (Proteintech, 81115‐1‐RR, 1:8000, RRID: AB_2923704).

### 5.8 RT‐QPCR

RAW264.7 macrophages were seeded into 6‐well plates at a density of 100,000 cells per well and allowed to adhere overnight. To establish the foam cell model and evaluate the therapeutic effects, the cells were stimulated with ox‐LDL (100 µg/mL) and co‐incubated with PB, CuPB, or CuPB@HA nanozymes (100 µg/mL) for (24 h). Total RNA was extracted from the cultured cells using the RNAeasy Animal Total RNA Isolation Kit (Beyotime, R0027, Shanghai, China) according to the manufacturer's instructions. Subsequently, complementary DNA (cDNA) was synthesized using the HiScript II Q RT SuperMix (Vazyme, Nanjing, China). Quantitative real‐time PCR was performed on a LightCycler 480 II system (Roche) utilizing a SYBR Green‐based fluorescent assay. The amplification protocol included a standardized annealing temperature of 60°C for all primer pairs. All specific primer sequences used in this study are detailed in Table S1.

### 5.9 Immunofluorescence Staining

To visually assess the phenotypic polarization and the expression of lipid scavenger receptors, RAW264.7 macrophages were seeded into confocal imaging dishes at a density of 5,000 cells per dish and allowed to adhere overnight. To induce the pathogenic foam cell phenotype and evaluate the targeted therapeutic efficacy, the cells were stimulated with ox‐LDL (100 µg/mL) and simultaneously co‐incubated with PB, CuPB, or CuPB@HA nanozymes (100 µg/mL) for 24 h. Following the designated treatments, the cells were gently washed with PBS and fixed with 4% paraformaldehyde (PFA) for 20 min at room temperature. After rinsing three times with PBS, the cellular membranes were permeabilized using a 0.1% Triton X‐100 solution in PBS for 20 min. To prevent non‐specific antibody binding, the cells were blocked with 5% bovine serum albumin (BSA) for 1 h at room temperature. Subsequently, the samples were incubated overnight at 4°C with specific primary antibodies targeting the M1 marker iNOS (Proteintech, 22226‐1‐AP,1:400), the M2 marker ARG1 (Proteintech, 16001‐1‐AP, 1:400), or the lipid uptake receptor CD36 (MedChemExpress, HY‐P86458, 1:400). The next day, following thorough PBS washes, the cells were incubated with a green fluorescent secondary antibody (488‐Goat Anti‐Rabbit Recombinant Secondary Antibody (Proteintech, RGAR002, 1:400) for 1 h at room temperature in the dark. The cell nuclei were then counterstained with DAPI, and the specimens were preserved using an antifade mounting medium. Finally, high‐resolution fluorescence images were acquired using a confocal laser scanning microscope, and the relative fluorescence intensities were quantitatively analyzed using ImageJ software.

### 5.10 Animals And Ethics Statement

Male apolipoprotein E‐deficient (ApoE^−/−^) mice (RRID: IMSR_JAX:002052) (7 weeks old) and age‐matched wild‐type C57BL/6 mice (RRID: MGI:2159769) (used as healthy controls) were purchased from YiShang Co., Ltd., Shanghai, China. Prior to any experimental procedures, all mice were acclimatized for 7 days in a specific pathogen‐free (SPF) environment maintained at a constant temperature (22 ± 2°C) and humidity (55 ± 5%) under a standard 12 h light/dark cycle, with ad libitum access to standard laboratory chow and water. To establish the in vivo atherosclerosis model, the ApoE^−/−^ mice were transitioned to a high‐fat diet (HFD) for 12 weeks, with systemic nanozyme interventions initiated during the final 3 weeks of the HFD regimen.

All in vivo procedures were conducted in strict adherence to the guidelines established by the Institutional Animal Care and Use Committee (IACUC) of Shanghai Sixth People's Hospital (Shanghai, China). Prior ethical clearance for these studies was officially granted by both the Animal Ethics Committee of Shanghai Jiao Tong University and the Animal Experimental Welfare Ethics Committee of Shanghai Sixth People's Hospital (Approval No. 2024‐0827).

### 5.11 Histopathological And Immunohistochemical Analyses

At the end of the predefined treatment regimen, the mice were euthanized, and the whole aortas, aortic roots, and major organs (liver, spleen, lung, and kidney) were carefully excised and fixed. First, to evaluate the global atherosclerotic plaque burden, the entire aortas were longitudinally dissected and subjected to Oil Red O (ORO) staining. Subsequently, the isolated aortic roots were embedded in paraffin. These cross‐sections were stained with ORO to quantify localized lipid accumulation and Hematoxylin and Eosin (H&E) to evaluate the necrotic core area and overall plaque morphology. Masson's trichrome staining was additionally performed on adjacent sections to visualize intralesional collagen deposition and precisely assess plaque structural stability. Furthermore, immunohistochemical (IHC) staining was conducted on the aortic root sections using a specific primary antibody against CD36 (Proteintech, 18836‐1‐AP, 1:400) to analyze the in vivo expression of this key lipid scavenger receptor within the plaque microenvironment. Finally, to systematically assess the in vivo biosafety profile of the nanozymes, sections of the major metabolic and excretory organs were similarly stained with H&E to screen for any potential tissue toxicity, necrosis, or pathological alterations across all groups.

### 5.12 In Vivo Therapeutic Regimen and Magnetic Resonance Imaging (MRI)

Following the establishment of the atherosclerosis model, the HFD‐fed ApoE^−/−^ mice were randomly divided into five experimental cohorts: Control, Model (Saline), PB, CuPB, and CuPB@HA group. The mice were systemically administered the respective formulations via tail vein injection at a dose of 0.5 mg/kg (Fe). The therapeutic regimen consisted of injections given twice a week for a total of 5 treatments. To non‐invasively evaluate the therapeutic efficacy and meticulously monitor plaque morphology in the carotid arteries, in vivo MRI was performed on the mice at the experimental endpoint (the final day of treatment). Image acquisition was executed using a uMR 9.4 T MRI system (United Imaging Life Science Instrument, China) utilizing a fast spin‐echo (FSE) sequence synchronized with continuous respiratory triggering to minimize physiological motion artifacts. The specific high‐resolution scanning parameters were strictly maintained as follows: repetition time (TR) = 3000 ms; echo time (TE) = 23.04 ms; field of view (FOV) = 17 mm × 20 mm; slice thickness = 0.6 mm; and 10 slices per scan.

### 5.13 Single‐Cell RNA Sequencing (scRNA‐seq) Data Processing

Public scRNA‐seq data from human atherosclerotic plaques were retrieved from the GEO database (accession number: GSE253903). Data analysis was performed using the Seurat R V5. To ensure high data quality, strict filtering criteria were applied to the raw gene‐cell matrix: cells were retained only if they possessed a mitochondrial gene percentage < 30%, a total UMI count (nCount_RNA) < 40,000, and a number of detected genes (nFeature_RNA) between 200 and 6000.

Following quality control, the data were normalized, and the top 2,000 highly variable genes were identified for principal component analysis (PCA). Cell clustering was performed using the Shared Nearest Neighbor (SNN) modularity optimization‐based clustering algorithm, and the clusters were visualized via Uniform Manifold Approximation and Projection (UMAP).

### 5.14 Bulk RNA Sequencing and Functional Enrichment

For bulk transcriptomic profiling, total RNA was extracted from RAW264.7 macrophages in the Control, Model, and CuPB@HA‐treated groups, with three independent biological replicates per group. Raw sequencing reads were subjected to quality control using FastQC v0.12.1, and quality‐control reports were summarized using MultiQC v1.35. Reads were aligned to the mouse reference genome GRCm39 using HISAT2 v2.2.2, with gene annotation based on Ensembl release 114. The resulting alignment files were processed using Samtools v1.23.1, and gene‐level read counts were generated using featureCounts v2.1.1.

Differential expression analysis was performed in R v4.4.1 using edgeR v4.4.2. Raw counts were normalized using the trimmed mean of M‐values (TMM) method. Differentially expressed genes (DEGs) were identified using |fold change| > 1.5 and FDR < 0.05. Gene Ontology (GO) and KEGG pathway enrichment analyses were performed using clusterProfiler v4.14.6 with org.Mm.eg.db v3.20.0. Enriched terms were reported with the corresponding *P* values, adjusted *P* values, and *q*‐values.

To identify core regulatory nodes, a protein‐protein interaction (PPI) network was constructed by mapping the DEGs to the STRING database. The resulting network was imported into Cytoscape for topological analysis and visualization, and hub genes were identified based on their degree of connectivity.

## Author Contributions

**Jianliang Ou**: conceptualization, methodology, data curation, writing – original draft. **Qinglu Zang**: conceptualization, methodology. **Hongxu Zhu**: software, methodology. **Lingling Zhou**: methodology, data curation. **Junjie Cheng**: supervision, writing – review and editing, writing – original draft. **Yingliang Wei**: methodology, data curation. **Wenxian Du**: writing – review and editing, writing – original draft, project administration. **Di Yang**: investigation, methodology, data curation. **Mingkang Wang**: methodology, data curation, formal analysis. **Ruihua Ji**: conceptualization, methodology. **Yue Wu**: software, data curation. **Xiaojun Cai**: methodology, formal analysis. **An Zeng**: writing – review and editing, funding acquisition, project administration, supervision. **Yuehua Li**: supervision, project administration. **Jianrong Wu**: methodology, data curation, formal analysis.

## Conflicts of Interest

The authors declare no conflicts of interest.

## Supporting information




**Supporting File**: advs76976‐sup‐0001‐SuppMat.docx.

## Data Availability

The datasets generated for this study are available on request to the corresponding author.
