# Supplementary material for: Copper‐Doped Prussian Blue Nanozymes With Hyaluronic Acid‐Mediated Targeting Alleviate Oxidative Stress and Regulate Cholesterol Handling for Atherosclerosis Therapy
Source: Adv Sci (Weinh). 2026 Aug 5;13(60):e76976. doi: 10.1002/advs.76976 (PMC13440190; doi:10.1002/advs.76976)
Supplement: Supplementary file 1 [file ADVS-13-e76976-s001.docx]

**Supporting Information**

**Copper-Doped Prussian Blue Nanozymes with Hyaluronic Acid-Mediated Targeting Alleviate Oxidative Stress and Regulate Cholesterol Handling for Atherosclerosis Therapy**

Jianliang Ou^1†^, Ruihua Ji^2†^, Qinglu Zang^1,6†^, Mingkang Wang^7^, Lingling Zhou^1,8^, Yingliang Wei^9^, Di Yang^4^, Yue Wu^1,10^, Hongxu Zhu^1^, Jianrong Wu^5^, Xiaojun Cai^5^, Wenxian Du^1^, Junjie Cheng^3*^, An Zeng^4*^, Yuehua Li^1*^

1. Department of Radiology, Shanghai Jiao Tong University School of Medicine Affiliated Sixth People’s Hospital, Shanghai, 200233, People’s Republic of China;
2. Department of Anesthesiology, Changzheng Hospital, Naval Medical University, Shanghai 200003, China;
3. Department of Nutrition and Food Hygiene, School of Public Health, Southeast University, Nanjing, China
4. Key Laboratory of Multi-Cell Systems, Chinese Academy of Sciences Center for Excellence in Molecular Cell Science, Institute of Biochemistry and Cell Biology, Shanghai Institutes for Biological Sciences, University of the Chinese Academy of Sciences, Chinese Academic of Sciences, Shanghai 200031, China
5. Department of Ultrasound in Medicine, Shanghai Jiao Tong University School of Medicine Affiliated Sixth People’s Hospital, Shanghai, 200233, People’s Republic of China;
6. College of Chemistry and Materials Science, Shanghai Normal University Shanghai, 200233, People’s Republic of China;
7. Wuhan United Imaging Life Science Instruments Ltd., Wuhan, 430000, People’s Republic of China;
8. Faculty of Medical Imaging Technology, College of Health Science and Technology, Shanghai Jiao Tong University School of Medicine;
9. Shanghai Normal University College of Life Science, Shanghai, 200233, People’s Republic of China;
10. Department of Pharmacy, Renji Hospital, School of Medicine, Shanghai Jiao Tong University, Shanghai, 200127, People’s Republic of China;

† These authors contributed equally to the work.

*** Corresponding authors:**

Junjie Cheng: [jjcheng@seu.edu.cn](mailto:jjcheng@seu.edu.cn)

An Zeng: [azeng@sibcb.ac.cn](mailto:azeng@sibcb.ac.cn)

Yuehua Li: [liyuehua77@sjtu.edu.cn](mailto:liyuehua77@sjtu.edu.cn)

- **Supporting Figures**

**Figure S1.** Cell-type-specific transcript abundance of CD44. Violin plots comparing CD44 expression levels across 12 major cell types identified in human carotid plaques (GSE253903), stratified by Asymptomatic and Symptomatic groups. Internal boxplots represent the median and interquartile range. Statistical significance between clinical groups was determined by the Wilcoxon rank-sum test;

**Figure S2. Validation of CD44 expression in atherosclerotic lesions and ox-LDL-induced macrophage foam cells.** (A) Representative immunofluorescence images of CD44 expression in aortic root sections from C57BL/6 control mice and HFD-fed ApoE−/− mice from the Saline/Model group. DAPI was used for nuclear staining (scale bars: 100 μm). (B) Representative Western blot analysis of CD44 protein expression in control RAW264.7 macrophages and ox-LDL-induced foam cells. β-actin was used as the loading control. (C) Quantitative analysis of CD44 protein expression normalized to β-actin. Statistical significance was assessed using an unpaired two-tailed Student’s t-test. ***p* < 0.01. (D) Quantitative analysis of CD44 mean fluorescence intensity in aortic root sections. Data are presented as mean ± SD (n = 3). Statistical significance was assessed using an unpaired two-tailed Student’s t-test. ***p* < 0.01.

**Figure S3.** Expression density of functional gene signatures. UMAP-based feature density plots showing the global distribution of transcripts related to inflammation (IL1B, TNF, IL6, IL10), redox homeostasis (SOD1, NFE2L2), and lipid metabolism (CD36, ABCA1, ABCG1).

**Figure S4.** Co-expression of CD44 with pathogenic mediators. Joint density UMAP plots visualizing the integrated expression situation of CD44 and the functional markers detailed in (**Figure S2**), highlighting the co-localization within the macrophage lineage.

**Figure S5.** Transcriptional profiles of macrophage sub-clusters. Dot plot illustrating the expression of canonical and subtype-specific marker genes across the six annotated macrophage populations.

**Figure S6.** M1 markers (HLA-DRA, IL1B) across macrophage subtypes in Asymptomatic vs. Symptomatic conditions. Statistical significance was assessed via Wilcoxon rank-sum test; * *p* < 0.05, ** *p* < 0.01, *** *p* < 0.001, **** *p* < 0.0001; ns: no significant difference (*p* > 0.05).

**Figure S7.** M2 markers (CD163, MS4A4A) across macrophage subtypes in Asymptomatic vs. Symptomatic conditions. Statistical significance was assessed via Wilcoxon rank-sum test; **p* < 0.05, ** *p* < 0.01, *** *p* < 0.001, **** *p* < 0.0001; ns: no significant difference (*p* > 0.05).

**Figure S8. TEM characterization of PB and CuPB nanozymes.** (A) TEM image of PB nanoparticles showing the typical cubic morphology (scale bar: 50 nm). (B) TEM image of CuPB nanoparticles after copper doping, showing that the nanocubic morphology was largely maintained (scale bar: 50 nm)

**Figure S9. XRD characterization of PB and CuPB@HA nanozymes.** (A) XRD pattern of PB nanoparticles. (B) XRD pattern of CuPB@HA nanozymes. The retained characteristic diffraction peaks indicate that HA coating did not disrupt the PB-like crystalline framework of the CuPB core.

**Figure S10.** **XPS characterization of PB and CuPB@HA nanozymes.** (A) High-resolution Fe 2p XPS spectrum of PB nanoparticles. (B) High-resolution Cu 2p XPS spectrum of CuPB@HA nanozymes, showing the coexistence of Cu²⁺ and Cu⁺ species.

**Figure S11.** FT-IR characterization of HA, PB, CuPB, and CuPB@HA.

**Figure S12.** Thermogravimetric analysis of CuPB and CuPB@HA.

**Figure S13.** UV–vis absorption spectra of ABTS^+^· in the presence of CuPB@HA at different concentrations.

**Figure S14.** UV–vis absorption spectra of DPPH· in the presence of CuPB@HA at different concentrations.

**Figure S15.** ESR spectra of ·OH signals in the presence of CuPB@HA at different concentrations.

**Figure S16.** Concentration-dependent SOD-like activity of CuPB@HA. (A) ESR spectra showing the concentration-dependent attenuation of ·O_2_^-^ signals after CuPB@HA treatment. (B) Concentration-dependent SOD-like activity of CuPB@HA measured using a SOD assay kit.

**Figure S17. Cytocompatibility of CuPB and CuPB@HA in RAW264.7 and THP-1 macrophage cells.** (A) CCK-8 assay showing the viability of RAW264.7 macrophages after incubation with different concentrations of CuPB or CuPB@HA for 24 h. (B) CCK-8 assay showing the viability of THP-1 macrophage cells after incubation with different concentrations of CuPB or CuPB@HA for 24 h. Both CuPB and CuPB@HA showed good cytocompatibility within the tested concentration range of 0–200 μg/mL. Data are presented as the mean ± SD (n=3).

**Figure S18.** Quantitative analysis of ABCA1, CD36, ABCG1, iNOS, ARG1, NRF2, SOD1, IL-1β, TNF-α, IL-10, and IL-6 protein expression levels normalized to GAPDH. Data are presented as mean ± SD (n=3). Statistical significance was assessed via one-way ANOVA (**p* < 0.05, ** *p* < 0.01, *** *p* < 0.001, **** *p* < 0.0001).

**Figure S19.** Regulatory effects of CuPB@HA on lipid metabolism and inflammatory polarization in THP-1-derived macrophage-like cells.

**Figure S20. Concentration-dependent inhibition of ox-LDL-induced lipid accumulation by CuPB@HA nanozymes in RAW264.7 macrophages.** (A) Representative Oil Red O (ORO) staining images of RAW264.7 macrophages after treatment with different concentrations of CuPB@HA (scale bars: 50 μm. (B) Quantitative analysis of the relative lipid droplet area based on ORO staining. RAW264.7 macrophages were pre-stimulated with ox-LDL (100 μg/mL) for 24 h and then treated with CuPB@HA at different concentrations ranging from 3.125 to 200 μg/mL. Data are presented as mean ± SD (n = 3). Statistical significance was assessed by one-way ANOVA followed by Tukey’s post hoc test. ns, not significant.

**Figure S21.** Data normalization of the bulk RNA-sequencing dataset. Box plots illustrating the global distribution of gene expression levels across all individual samples before (left) and after (right) normalization and batch effect correction. The samples comprise three biological replicates for each experimental cohort: Control (C1-C3), Model (M1-M3), and Treat (T1-T3).

**Figure S22.** Principal component analysis (PCA) of the transcriptomic profiles. 2D PCA score plot evaluating the global gene expression patterns of macrophages from the Control (Con), Model, and CuPB@HA-treated (Treat) groups.

**Figure S23.** Transcriptional profiling of functional lipid metabolism-related modules.

**Figure S24. Immunofluorescence validation of plaque stability-associated features *in vivo*.** (A) Representative MMP9 immunofluorescence staining images of aortic root sections and corresponding quantification of mean fluorescence intensity. (B) Representative α-SMA immunofluorescence staining images of aortic root sections and corresponding quantification of mean fluorescence intensity. Control indicates wild-type C57BL/6 mice; Saline and CuPB@HA indicate HFD-fed ApoE−/− mice treated with saline or CuPB@HA, respectively. DAPI was used for nuclear staining (scale bars: 100 μm) Data are presented as mean ± SD (n = 3). Statistical significance was assessed by one-way ANOVA followed by Tukey’s post hoc test. ns, not significant; **p* < 0.05, ***p* < 0.01, ****p* < 0.001.

**Figure S25.** Anatomical localization for in vivo magnetic resonance imaging (MRI) of the carotid arteries. The red dashed line denotes the precise anatomical slice.

**Figure S26. Body weight monitoring during the in vivo study.**

(A) Body weight curves of mice in each group throughout the experimental period. (B) Percentage body weight change relative to the initial body weight. No animal in any group exhibited body weight loss exceeding 15% of its initial body weight at any time point during the study. Data are presented as mean ± SD (n=8).

**Figure S27. Serum liver function indicators after different treatments.** Serum levels of (A) aspartate aminotransferase (AST), (B) alanine aminotransferase (ALT), and (C) alkaline phosphatase (ALP) in mice from the Control, Saline, PB, CuPB, and CuPB@HA groups. Data are presented as mean ± SD, n = 6. Statistical analysis was performed using one-way ANOVA followed by Tukey’s post hoc test. ns, not significant.

**Figure S28. Serum kidney function indicators after different treatments.** Serum levels of (A) blood urea nitrogen (BUN), (B) urea, and (C) uric acid (UA) in mice from the Control, Saline, PB, CuPB, and CuPB@HA groups. Data are presented as mean ± SD, n = 6. Statistical analysis was performed using one-way ANOVA followed by Tukey’s post hoc test. ns, not significant.

**Figure S29. Hematological parameters after different treatments.** Blood routine analysis of (A) red blood cells (RBC), (B) white blood cells (WBC), (C) hemoglobin (HGB), and (D) platelets (PLT) in mice from the Control, Saline, PB, CuPB, and CuPB@HA groups. Data are presented as mean ± SD, n = 6. Statistical analysis was performed using one-way ANOVA followed by Tukey’s post hoc test. ns, not significant.

**Figure S30. Preliminary validation of LXR-related signaling in CuPB@HA-mediated regulation of macrophage cholesterol-handling mediators.** Western blot analysis and corresponding densitometric quantification of ABCA1, CD36, PPARγ, and LXRα protein expression in RAW264.7 macrophages after different treatments. Cells were stimulated with ox-LDL and treated with CuPB@HA in the presence or absence of the LXR antagonist GSK2033. β-actin was used as the loading control. Protein expression levels were normalized to β-actin. Significance was assessed via one-way ANOVA with Tukey’s post hoc test (n=3)(**p* < 0.05, ** *p* < 0.01, *** *p* < 0.001, **** *p* < 0.0001; ns: no significant difference (*p* > 0.05).).

- **Tables**

**Table S1. Primer sequences used for RT-qPCR**

| Gene name | Species | | | Forward (5'-3') | Reverse (5'-3') |
| --- | --- | --- | --- | --- | --- |
| Cd36 | | mouse | ATGGGCTGTGATCGGAACTG | | GTCTTCCCAATAAGCATGTCTCC |
| Abca1 | | mouse | AAAACCGCAGACATCCTTCAG | | CATACCGAAACTCGTTCACCC |
| Abcg1 | | mouse | CTTTCCTACTCTGTACCCGAGG | | CGGGGCATTCCATTGATAAGG |
| Nos2 | | mouse | CAGGAGGAGAGAGATCCGATTTA | | GCATTAGCATGGAAGCAAAGA |
| Arg1 | | mouse | CTCCAAGCCAAAGTCCTTAGAG | | AGGAGCTGTCATTAGGGACATC |
| Il10 | | mouse | GCTCTTACTGACTGGCATGAG | | CGCAGCTCTAGGAGCATGTG |
| Il1b | | mouse | GCAACTGTTCCTGAACTCAACT | | ATCTTTTGGGGTCCGTCAACT |
| Tnf | | mouse | AAGCAAGCAGCCAACCAG | | TCTTCTGCCAGTTCCACG |
| Il6 | | mouse | TAGTCCTTCCTACCCCAATTTCC | | TTGGTCCTTAGCCACTCCTTC |
| Sod1 | | mouse | CAGGACCTCATTTTAATCCTCAC | | TGCCCAGGTCTCCAACAT |
| Nfe2l2 | | mouse | GCCCACATTCCCAAACAAGAT | | CCAGAGAGCTATTGAGGGACTG |
| β-actin | | mouse | GTCCCTCACCCTCCCAAAAG | | GCTGCCTCAACACCTCAACCC |
|  | |  |  | |  |

**Table S2. GO biological process enrichment results corresponding to Figure 5B,D.**

| Figure panel | ID | pvalue | p.adjust | qvalue |
| --- | --- | --- | --- | --- |
| Figure 5B | GO:1905954 | 0.000000533 | 0.00107825 | 0.00082627 |
|  | GO:0010876 | 0.00000062 | 0.00107825 | 0.00082627 |
|  | GO:1905952 | 0.000000974 | 0.00107825 | 0.00082627 |
|  | GO:0032611 | 0.00000147 | 0.00107825 | 0.00082627 |
|  | GO:0032651 | 0.00000147 | 0.00107825 | 0.00082627 |
|  | GO:0006869 | 0.00000479 | 0.00200639 | 0.00153751 |
|  | GO:0030301 | 0.0000357 | 0.00545816 | 0.00418262 |
|  | GO:0032640 | 0.00181078 | 0.03496712 | 0.02679553 |
|  | GO:0007249 | 0.00288787 | 0.04263356 | 0.03267038 |
|  | GO:0032635 | 0.00306712 | 0.04361797 | 0.03342474 |
| Figure 5D | GO:0006869 | 0.00000254 | 0.0086854 | 0.0072425 |
|  | GO:0010876 | 0.00000536 | 0.00918675 | 0.00766056 |
|  | GO:0010743 | 0.0000157 | 0.01317946 | 0.01098997 |
|  | GO:0010742 | 0.0000263 | 0.01317946 | 0.01098997 |
|  | GO:0008203 | 0.00013409 | 0.02175158 | 0.01813801 |
|  | GO:0050727 | 0.00015882 | 0.02175158 | 0.01813801 |
|  | GO:0050729 | 0.00021779 | 0.02175158 | 0.01813801 |
|  | GO:0032757 | 0.00055156 | 0.03047817 | 0.02541486 |
|  | GO:0032637 | 0.00146132 | 0.04865389 | 0.04057107 |
|  | GO:0032677 | 0.00146132 | 0.04865389 | 0.04057107 |
